# Maternal dietary patterns as predictors of neonatal body composition in Ethiopia: the IABC birth cohort study

**DOI:** 10.1186/s12884-025-07256-1

**Published:** 2025-04-02

**Authors:** Daniela Viktoria Nickel, Rasmus Wibaek, Henrik Friis, Jonathan C. K. Wells, Tsinuel Girma, Pernille Kaestel, Kim F. Michaelsen, Bitiya Admassu, Mubarek Abera, Matthias B. Schulze, Ina Danquah, Gregers S. Andersen

**Affiliations:** 1 Department of Molecular Epidemiology, German Institute of Human Nutrition Potsdam- Rehbruecke, Nuthetal, Germany; 2 Institute of Nutritional Science, University of Potsdam, Nuthetal, Germany; 3 NutriAct - Competence Cluster Nutrition Research Berlin-Potsdam, Nuthetal, Germany; 4 Department of Nutrition, Exercise and Sports, University of Copenhagen, Copenhagen, Denmark; 5 Clinical Epidemiology, Steno Diabetes Center Copenhagen, Gentofte, Denmark; 6 Population Policy and Practice Research and Teaching Department, UCL Great Ormond Street Institute of Child Health, London, UK; 7 Department of Pediatrics and Child Health, Jimma University, Jimma, Ethiopia; 8 Jimma University Clinical and Nutrition Research Partnership (JUCAN), Jimma University, Jimma, Ethiopia; 9 Department of Population and Family Health, Jimma University, Jimma, Ethiopia; 10 Department of Psychiatry, Jimma University, Jimma, Ethiopia; 11 Heidelberg Institute of Global Health, Universitaetsklinikum Heidelberg, Heidelberg, Germany

**Keywords:** Dietary patterns, Dietary diversity, Ethiopia, Neonate, Body composition, Pregnancy, iABC

## Abstract

**Background:**

Malnutrition during pregnancy is associated with adverse birth outcomes, but the importance of maternal diet during pregnancy for neonatal body composition remains inconclusive. This study investigated the role of maternal diet during pregnancy for neonatal body composition in the Ethiopian iABC birth cohort.

**Methods:**

The data stemmed from the first visit at birth comprising 644 mother-child pairs. Shortly after delivery, the diet of the last week of pregnancy was assessed by a non-quantitative and non-validated 18-items food frequency questionnaire. Multiple imputation was used to handle missing data. Twin births and implausible values were excluded from analysis (*n* = 92). The Dietary Diversity Score (0–9 points) was constructed and exploratory dietary patterns were derived via principal component analysis. Neonatal fat mass and fat-free mass were assessed by air-displacement plethysmography. The associations of maternal Dietary Diversity Score and exploratory dietary patterns with gestational age, neonatal anthropometric measures and body composition were investigated using multiple-adjusted linear regression analysis.

**Results:**

In this cohort (*n* = 552), mean ± standard deviation (SD) mother’s age was 24.1 ± 4.6 years and the median maternal Dietary Diversity Score was 6 (interquartile range = 5–7). An ‘Animal-source food pattern’ and a ‘Vegetarian food pattern’ were identified. The mean ± SD birth weight was 3096 ± 363 g and gestational age was 39.0 ± 1.0 weeks. Maternal adherence to the Animal-source food pattern, but not Vegetarian food pattern, was related to birth weight [79.5 g (95% confidence interval (CI): -14.6, 173.6)]. In the adjusted model, adherence to the Animal-source food pattern was associated with higher neonatal fat-free mass [53.1 g (95% CI: -20.3, 126.6)], while neonates of women with high compared to low adherence to Dietary Diversity Score and Vegetarian food pattern had higher fat mass [19.4 g (95% CI: -7.4, 46.2) and 33.5 g (95% CI: 2.8, 64.1), respectively].

**Conclusions:**

In this Ethiopian population, maternal diet during pregnancy was associated with neonatal body composition. The analysis of body composition adds important detail to the evaluation of maternal dietary habits for the newborn constitution.

**Supplementary Information:**

The online version contains supplementary material available at 10.1186/s12884-025-07256-1.

## Background

Despite substantial progress, maternal and child survival and health remain major public health concerns in sub-Saharan Africa (SSA) [1, 2]. Among various intrauterine factors, maternal diet is one of the major environmental factors influencing embryonic development and foetal growth [3], and maternal undernutrition has been shown to drive adverse pregnancy outcomes such as low birth weight (< 2500 g) [4].

Although a positive relationship between maternal adherence to a healthy diet and birth outcomes has been observed in high-income countries, data is still inconclusive [5]. Few studies have reported the association of the overall maternal diet with birth outcomes in Ethiopia or other SSA countries, and the results have been inconsistent [6, 7]. Although no association of a diversified diet of the mother with preterm delivery was observed in a Ghanaian population [7], Ethiopian mothers with higher dietary diversity during pregnancy were at lower risk of delivering preterm babies compared to those with lower dietary diversity [6]. A positive relation between maternal dietary diversity and birth weight was identified in SSA [6–8].

The literature investigating maternal nutrition and birth outcomes is dominated by studies of the intake of single nutrients or specific food groups in isolation [9–12]. Undernutrition is usually not caused by a lack of single micronutrients or macronutrients but is rather a result of multiple nutrient deficiencies [10]. Diets represent a complex exposure and are not easily explained by isolated nutrients [13]. These complex combinations of nutrients interacting with each other cannot be considered, when investigating singular nutrients or foods. An increasing number of epidemiological studies have applied dietary pattern analysis, which aims to examine the effect of the overall diet including foods, food groups and nutrients, their combination and variety as well as their frequency and quantity of intake [13, 14]. Despite the strength of dietary patterns to account for the complexity of nutritional behaviour, both hypothesis-driven and exploratory approaches have rarely been used for the investigation of the relation between maternal diet and birth outcomes in SSA.

The accurate assessment of neonatal nutritional status remains a public health concern. Traditional measures such as birth weight and length still serve as common indicators of foetal undernutrition [15]. These conventionally assessed birth outcomes consider the body as a single compartment and may not sufficiently measure nutritional status and later disease risk [16]. The assessment of neonatal body composition in addition to conventional birth outcomes may yield more informative results on the child’s health status. Most studies in low- and middle-income countries rely on neonatal anthropometry when examining body composition [17]. Recently, air-displacement plethysmography (ADP) has been proven to precisely and accurately measure body composition of infants in diverse settings, including Ethiopia [18], and may be used as a more accurate outcome measure when investigating the impact of maternal diet on the offspring`s nutritional status.

The present work aimed to investigate the effect of maternal dietary diversity and adherence to exploratory dietary patterns during pregnancy on neonatal body composition and anthropometrics among mother-child pairs from semi-urban Ethiopia.

## Methods

### Study design and population

The detailed primary objectives and design of the prospective birth cohort study *infant anthropometry and body composition study* (iABC) can be found elsewhere [18–20]. Women who gave birth at term (gestational age ≥ 37 weeks) to an infant ≥ 1500 g without congenital malformations at Jimma University Specialized Hospital (JUSH) between December 2008 and October 2012, who were 15–45 years old and planned to stay in Jimma for at least 6 months after delivery were invited to participate in the study. Mother-child pairs were examined within 48 h after delivery by trained research nurses. The present data stemmed from a subset focused on the first visit at birth comprising 644 mother-child pairs (Supplementary Fig. 1, Additional File 1). The study was conducted based on the guidelines developed by the Declaration of Helsinki, and ethical approval was obtained from the Jimma University Ethical Review Committee (reference number 23/12/2008). The study was registered at the International Standard Randomised Controlled Trial Number register with the registry number ISRCTN46718296. Every mother gave written informed consent on behalf of herself and her newborn [18–20].

### Dietary assessment

Dietary data were obtained by an Ethiopian-specific, non-quantitative and non-validated food frequency questionnaire (FFQ), which comprised 18 food items as presented in Supplementary Table 1, Additional File 1. The FFQ reflected the past 7 days. At the first visit shortly after delivery, the past week represented the last week of pregnancy. The participants could choose between four different answering options: “not at all”, “one to two days”, “three to six days” and “every day”. They answered the questionnaire together with nurses, who could speak both local languages Amharic and Afan Oromo as well as English.

Different statistical approaches are used to characterise dietary patterns. Driven by a hypothesis and constructed based on previous scientific evidence, dietary indices are an a priori approach [14]. As an example, the Dietary Diversity Score (DDS) aims to reflect nutrient adequacy of the diet, including food groups tailored towards this purpose [21]. According to the Women’s Dietary Diversity Score proposed by the United Nation`s Food and Agricultural Organisation (FAO), the DDS represents a valid, simple and rapid proxy indicator of dietary quality [21, 22].

In contrast, the *a posteriori* approach derives dietary patterns of available dietary data at hand using statistical modelling. Principal component analysis (PCA) is a common statistical technique to identify patterns of foods that are likely consumed together in the population [23].

### Assessment of birth outcomes

The neonates were physically examined at birth providing measures of gestational age (in weeks), length (in cm), weight (in g), fat mass (FM) (in g) and fat-free mass (FFM) (in g). Gestational age was determined using the New Ballard Score, which provides a valid and accurate method of clinically assessing gestational age in newborns, and is based on neuromuscular and physical maturity [24, 25]. Birth length was determined in duplicate to the nearest 0.1 cm using a SECA 416 Infantometer (SECA, Hamburg, Germany). An infant air-displacement plethysmograph, PEA POD^®^ (Life Measurements, Concord, CA, USA) was used to measure birth weight to the nearest 0.0001 kg, and to assess neonatal FM and FFM, which has previously been described in more detail [19, 20, 26–28]. The PEA POD^®^ has recently been identified to precisely and accurately measure body composition of infants, even in Ethiopian settings [18]. Absolute FM and FFM were adjusted for body size by dividing by length squared to give the fat mass index (FMI in kg/m^2^) and fat-free mass index (FFMI in kg/m^2^) [16].

### Assessment of covariates

General, obstetric and socio-demographic information were given by an interview-based questionnaire performed by trained study nurses. Mother’s age (years), date of delivery, sex of the child (male/female), number of antenatal care visits, parity (number of pregnancies ≥ 20 weeks of gestation), outcome of pregnancy (singleton/twin/triplet), mode of delivery (spontaneous vertex delivery/breech/instrumental vaginal delivery/caesarean section/other), delivery complications (no/premature rupture of membranes, chorioamnionitis/prolonged first stage of labour/prolonged second stage of labour/antepartum haemorrhage/postpartum haemorhage/foetal distress), and supplement (yes/no) and medication intakes (yes/no) during pregnancy were collected. Moreover, information about diseases (yes/no), religious background (Muslim/Orthodox Christian/Protestant/Catholic/other) and parental socioeconomic characteristics (Supplementary Table 2, Additional File 1) were obtained. Maternal weight was measured to the nearest 0.1 kg by Tanita 418 Bioimpedance analyser (Tanita Corp., USA). The mother’s height was determined to the nearest 0.1 cm using a SECA 214 stadiometer (SECA, Hamburg, Germany). The postpartum BMI of the mother was calculated using the mother’s weight (kg) and dividing it by the squared mother’s height (m).

All collected data were double-entered in EpiData Software Version 3 (EpiData Association, Odense, Denmark) by two trained data clerks.

### Statistical analyses

Twin births were excluded from subsequent analyses (*n* = 18). According to the reference data for FM and FFM from birth to the age of six months in the semi-urban Ethiopian population proposed by Andersen et al. [18], absolute FFM and FM were out of the reference range in 92 (15%) and 45 (7%) neonates in the 626 mother-child pairs, respectively. The reference ranges were defined as FFM below 2.09 kg or above 3.30 kg and FM below 0.03 kg or above 0.49 kg in girls, and FFM below 2.27 kg or above 3.43 kg and FM below 0.01 kg or above 0.53 kg in boys, respectively [18]. Values out of the reference range were assumed to be implausible and recoded into missing values. Missing variables as presented in Supplementary Table 3, Additional File 1, were filled in using multiple imputation techniques (Supplementary Statement 1, Additional File 1). Some observations (*n* = 74) of imputed and recalculated FFMI and FMI were excluded because they remained outside the reference range proposed by Andersen et al., which were defined as FFMI below 9.6 kg/m² or above 13.2 kg/m² and FMI below 0.13 kg/m² or above 2.01 kg/m² in girls, and FFMI below 10.03 kg/m² or above 13.57 kg/m² and FMI below 0.02 kg/m² or above 2.15 kg/m² in boys, respectively [18]. The resulting analytical sample included 552 participants (Supplementary Fig. 1, Additional File 1).

Dietary data was checked for plausibility by investigating the intake distribution of the 18 food items in comparison to Ethiopian food group consumption identified in the Ethiopian National Food Consumption Survey [29] For the DDS construction, the 18 food items were collapsed into 9 food groups referred to as DDS categories (Supplementary Table 1, Additional File 1). The aggregation was based on the guidance on assigning individual foods to food groups [21]. A mother who consumed a food group at least once in the last week was scored as one, otherwise she was coded as zero. The individual’s DDS was obtained by the sum of food groups consumed during the last week. The maximum achievable DDS score was nine.

The 18 original food items were collapsed into 13 variables according to nutrient profile and culinary use to derive exploratory dietary patterns via PCA (Supplementary Table 1, Additional File 1). Orthogonal (varimax) rotation was applied to generate uncorrelated and interpretable principal components, explaining the maximum of total variance in the dietary data [30, 31]. Three criteria were used to decide, how many principal components to retain: the inspection of the scree plot, an eigenvalue greater than one, and the plausibility and interpretability of the principal components [30]. The latter criterium was fulfilled, when principal components were characterised by at least three food variables with large factor loadings (≥ 0.35). The achieved individual factor scores facilitated the ranking of participants according to the degree to which they adhered to each dietary pattern [31].

Both the DDS and the dietary pattern scores were divided into three groups approximately equal in size using the tertiles as borders, referred to as first, second and third tertile, which represented lower, moderate and higher adherence to the dietary pattern, respectively. General and socio-demographic characteristics were examined across the tertiles of the DDS and the pattern scores. For normally distributed continuous variables, means and standard deviations (SD) were calculated. Non-normally distributed continuous variables and the DDS were shown as medians and interquartile ranges (IQR), whereas categorical variables were depicted as relative frequencies.

Multiple linear regression analysis was applied to investigate the associations between the tertile-based groups of DDS and exploratory dietary patterns with continuous gestational age, birth weight, birth length and neonatal body composition (FFM, FM, FFMI, FMI). The main results focused on absolute FFM and FM, while the results for FFMI and FMI are presented in the supplement. Mean differences (β) and 95% confidence intervals (CI) were calculated for the second and third tertiles of the dietary patterns, using the first tertile as the reference group. Three different models were constructed to account for possible confounders and covariates, which were chosen based on the literature and considered to be associated with either the main outcome and the exposure or the outcome only [4, 11, 32, 33]: Model 1 was adjusted for age of the mother, gestational age and sex of the child. In addition to the covariates included in model 1, model 2 was also adjusted for parity, mode of delivery, delivery complications, supplementation and medication during pregnancy, number of antenatal care visits and diseases. Model 3 was further adjusted for socioeconomic variables including the possession of consumer durables, access to electricity and private piped water as well as both mother’s and father’s occupation and education. The exploratory dietary patterns were additionally adjusted for the DDS, because positive correlations were previously identified between exploratory dietary patterns and the DDS [34]. Several sensitivity analyses were conducted (Supplementary Statement 2, Additional File 1). A significance level of 0.05 was used for all statistical tests and *p* values were corrected for the false discovery rate. However, due to the secondary objective and exploratory nature of the present work, the study results will be interpreted based on magnitude of effect size and clinical relevance rather than statistical significance. The statistical analyses were performed with SAS Version 9.4 Enterprise Guide Version 7.1 (SAS Institute Inc., NC, USA).

## Results

### Characteristics of the study population

In this cohort (mean mother’s age: 24.1 ± 4.6 years; 51.4% female children), the mothers were predominantly primiparous (54.6%), attended antenatal care (93.4%) and gave birth spontaneously (92.1%). The majority of mothers had a low socioeconomic status (low education: 60.4%; housewife: 61.1%). The neonatal mean weight was 3.1 ± 0.4 kg and gestational age was 39.0 ± 1.0 weeks. (Table 1)

**Table 1:** Descriptive characteristics across tertiles (T) of Dietary Diversity score among 552 Ethiopian mother-child pairs

		Dietary Diversity Score		
	*N* total	T1	T2	T3
n	552	210	152	190
**Household characteristics**				
Dietary Diversity Score [median (IQR)]	6 (5–7)	5 (4–5)	6 (6–6)	7 (7–8)
Animal-source food pattern score	0.00 (1.00)	-0.58 (0.60)	-0.00 (0.90)	0.65 (1.03)
Vegetarian food pattern score	0.00 (1.00)	-0.26 (0.87)	0.05 (1.07)	0.25 (1.01)
Mother’s age (years)	24.1 ± 4.6	24.2 ± 4.8	23.5 ± 4.7	24.5 ± 4.3
Mother’s body mass index (kg/m^2^)	22.6 ± 3.0	22.3 ± 2.7	22.5 ± 2.9	23.0 ± 3.2
**Obstetric history**				
Antenatal care (yes; %)	93.4	90.2	93.4	96.8
Parity (≥ 3; %)	21.9	24.1	22.4	19.2
Spontaneous vertex delivery (yes; %)	92.1	91.4	93.3	91.9
Delivery complications (no; %)	94.1	92.6	96.7	93.6
Diseases (yes; %)	4.2	5.1	4.3	3.1
Supplementation (yes; %)	13.9	7.1	15.5	20.0
Medication (yes; %)	15.5	11.1	17.1	19.0
**Religion**				
Muslim (%)	44.3	39.9	49.7	44.7
Orthodox Christianity (%)	37.8	43.5	35.1	33.5
**Mother’s education**				
Higher education (%)	14.0	7.8	13.7	21.0
**Father’s education**				
Higher education (%)	20.6	12.6	18.8	30.9
**Mother’s occupation**				
Employee (private and public; %)	20.3	16.8	16.5	27.2
**Father’s occupation**				
Employee (private and public; %)	59.5	57.7	59.9	61.3
**Consumer durables**				
Access to electricity (yes; %)	95.1	93.0	95.7	97.1
Access to private piped water (yes; %)	60.9	52.1	62.9	69.1
Possession of household items (> 5; %)	13.7	7.0	16.6	18.8
International wealth index	51.6 ± 18.3	45.8 ± 18.8	53.1 ± 17.9	57.0 ± 15.9
**Newborns**				
Gestational age (weeks)	39.0 ± 1.0	38.9 ± 1.1	39.0 ± 1.0	39.1 ± 1.1
Sex of the child (male; %)	48.6	48.9	49.7	47.4
**Anthropometric characteristics**				
Birth weight (g)	3095.6 ± 362.6	3076.9 ± 371.8	3079.3 ± 350.9	3129.5 ± 359.4
Length (cm)	49.4 ± 1.8	49.3 ± 1.8	49.3 ± 1.8	49.6 ± 1.8
**Body composition**				
Fat-free mass (g)	2845.8 ± 281.2	2836.9 ± 288.9	2847.8 ± 267.8	2854.0 ± 283.2
Fat-free mass index (kg/m^2^)	11.7 ± 0.7	11.7 ± 0.8	11.7 ± 0.8	11.6 ± 0.7
Fat mass (g)	236.7 ± 119.3	225.4 ± 115.3	225.5 ± 125.7	258.3 ± 115.4
Fat mass index (kg/m^2^)	1.0 ± 0.5	0.9 ± 0.5	0.9 ± 0.5	1.1 ± 0.5

Data were shown as mean ± standard deviation, unless otherwise stated. Analytical sample (N total = 552) excluded twin births (*n* = 18) and implausible values for Fat-free mass index and Fat mass index (*n* = 74). IQR = interquartile range

### Dietary diversity and exploratory dietary patterns

The median DDS was 6 (IQR: 5–7) and DDS tertiles ranged from 1 to 5 (first tertile), 6 (second tertile) and 7–9 (third tertile). (Table 1). Starchy staple foods were universally consumed, followed by legumes, green leafy vegetables and other fruits. In contrast, organ meat, milk and milk products and eggs were least frequently consumed (Supplementary Fig. 2, Additional File 1).

**Fig. 1:**
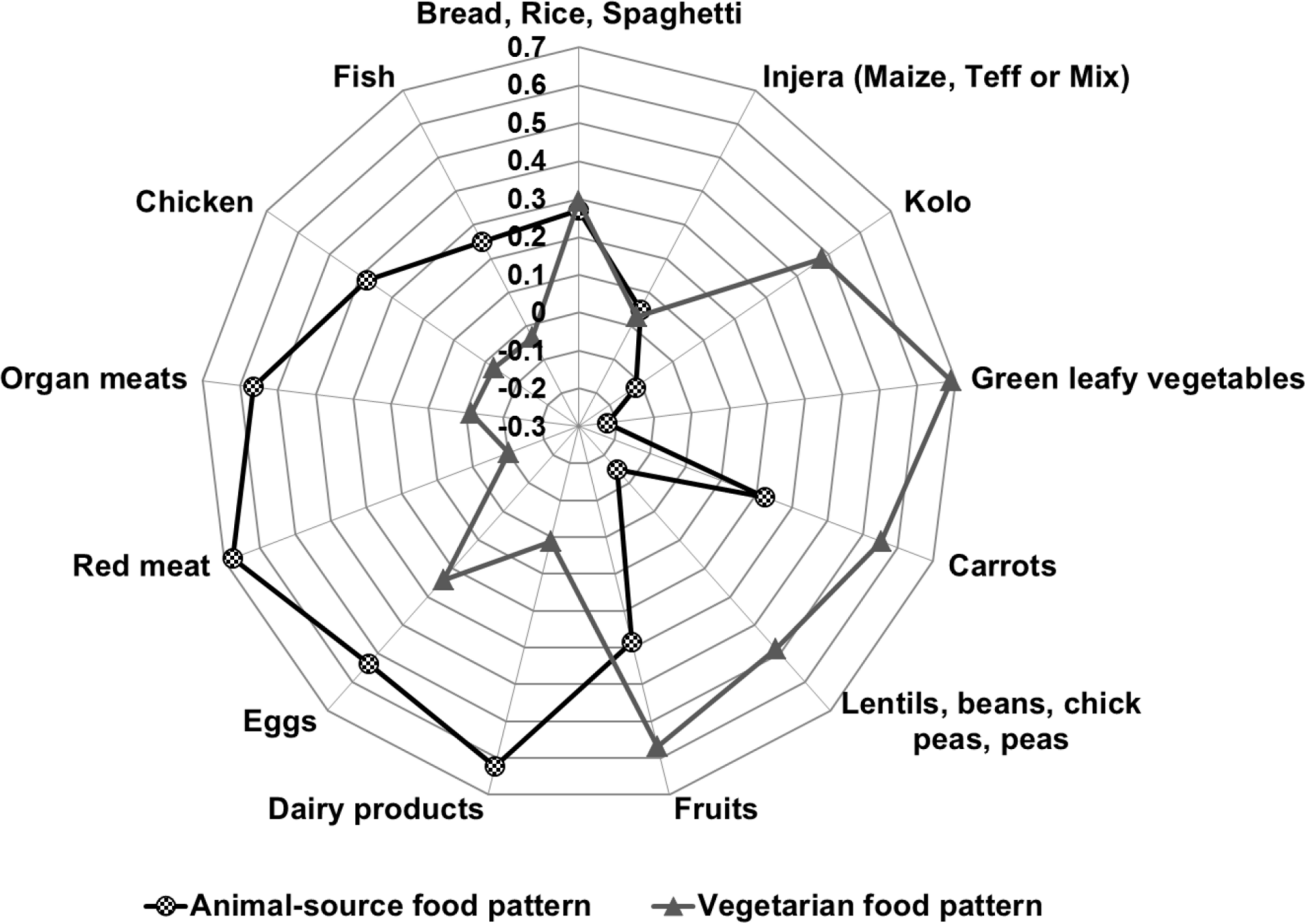
Dietary patterns derived by principal component analysis and rotated factor loadings in 552 pregnant Ethiopian women. Analytical sample (N total = 552) excluded twin births (*n* = 18) and implausible values for Fat-free mass index and Fat mass index (*n* = 74)

Two dietary patterns accounting for 28.0% of the total variance in food intake were identified (Fig. 1). The first identified pattern, called ‘Animal-source food pattern’ (AFP), explained 15.5% of the total variance in food intake. Meat, dairy, organ meat, eggs and chicken correlated positively with this pattern. The second ‘Vegetarian food pattern’ (VFP) explained 12.5% of the total variance in food intake and was mainly characterised by high intakes of vegetables, fruits, legumes and roasted grain snacks.

### Parental characteristics according to dietary patterns

The parental characteristics across the tertiles of the DDS are presented in Table 1. Compared to the first tertile, women in the third tertile had higher BMI and were more likely to receive antenatal care, and to take supplements or medications during pregnancy. They were of higher educational and occupational levels with greater access to electricity and private piped water. While the general parental characteristics were similarly distributed across the tertiles of the AFP compared to the distribution across the tertiles of the DDS, the distribution of parental characteristics across the tertiles of the VFP was different (Supplementary Table 4, Additional File 1).

### Dietary diversity, exploratory dietary patterns and birth outcomes

Compared to the first tertile of the DDS, babies born to mothers of the third tertile were of similar gestational age, birth weight and FFM, but had higher absolute FM (Table 1). Neonatal characteristics across the tertiles of the AFP and VFP scores are presented in Supplementary Table 5, Additional File 1.

**Table 2:** Association of Dietary Diversity Score with birth outcomes and body composition among 552 Ethiopian mother-child pairs

	Dietary Diversity Score		
	Mean difference (β) (95% confidence interval)		
	T1	T2	T3
n	210	152	190
**Gestational age (weeks)**			
Model 1	Ref.	0.12 (-0.11, 0.34)	0.22 (0.02, 0.43)
Model 2	Ref.	0.12 (-0.10, 0.35)	0.24 (0.02, 0.45)
Model 3	Ref.	0.11 (-0.12, 0.35)	0.19 (-0.04, 0.41)
**Birth weight (g)**			
Model 1	Ref.	-2.96 (-79.43, 73.51)	37.14 (-34.91, 109.18)
Model 2	Ref.	-1.77 (-79.43, 75.89)	35.28 (-38.48, 109.03)
Model 3	Ref.	-24.98 (-105.83, 55.88)	4.14 (-73.55, 81.83)
**Length (cm)**			
Model 1	Ref.	-0.03 (-0.43, 0.36)	0.22 (-0.15, 0.59)
Model 2	Ref.	-0.04 (-0.45, 0.36)	0.19 (-0.20, 0.57)
Model 3	Ref.	-0.17 (-0.58, 0.25)	0.05 (-0.35, 0.45)
**Fat-free mass (g)**			
Model 1	Ref.	7.18 (-51.12, 65.48)	7.42 (-48.27, 63.12)
Model 2	Ref.	4.38 (-54.69, 63.46)	3.99 (-52.78, 60.77)
Model 3	Ref.	-11.53 (-73.71, 50.65)	-14.13 (-74.69, 46.43)
**Fat mass (g)**			
Model 1	Ref.	-1.09 (-27.24, 25.06)	28.30 (4.17, 52.43)
Model 2	Ref.	2.34 (-24.78, 29.47)	28.81 (3.82, 53.80)
Model 3	Ref.	-2.90 (-31.11, 25.31)	19.40 (-7.38, 46.18)

Multiple-adjusted mean differences (β), 95% confidence intervals and *p* values were calculated by linear regression. Model 1: adjusted for age of the mother, sex of the child and gestational age (for outcome variables other than gestational age); model 2: model 1 + parity, mode of delivery, delivery complications, supplementation and medication during pregnancy, number of antenatal care visits and diseases; model 3: model 2 + socioeconomic variables (possession of consumer durables, access to electricity and private piped water, mother’s and father’s occupation and education). Analytical sample (N total = 552) excluded twin births (*n* = 18) and implausible values for Fat-free mass index and Fat mass index (*n* = 74). T = tertile

**Fig. 2:**
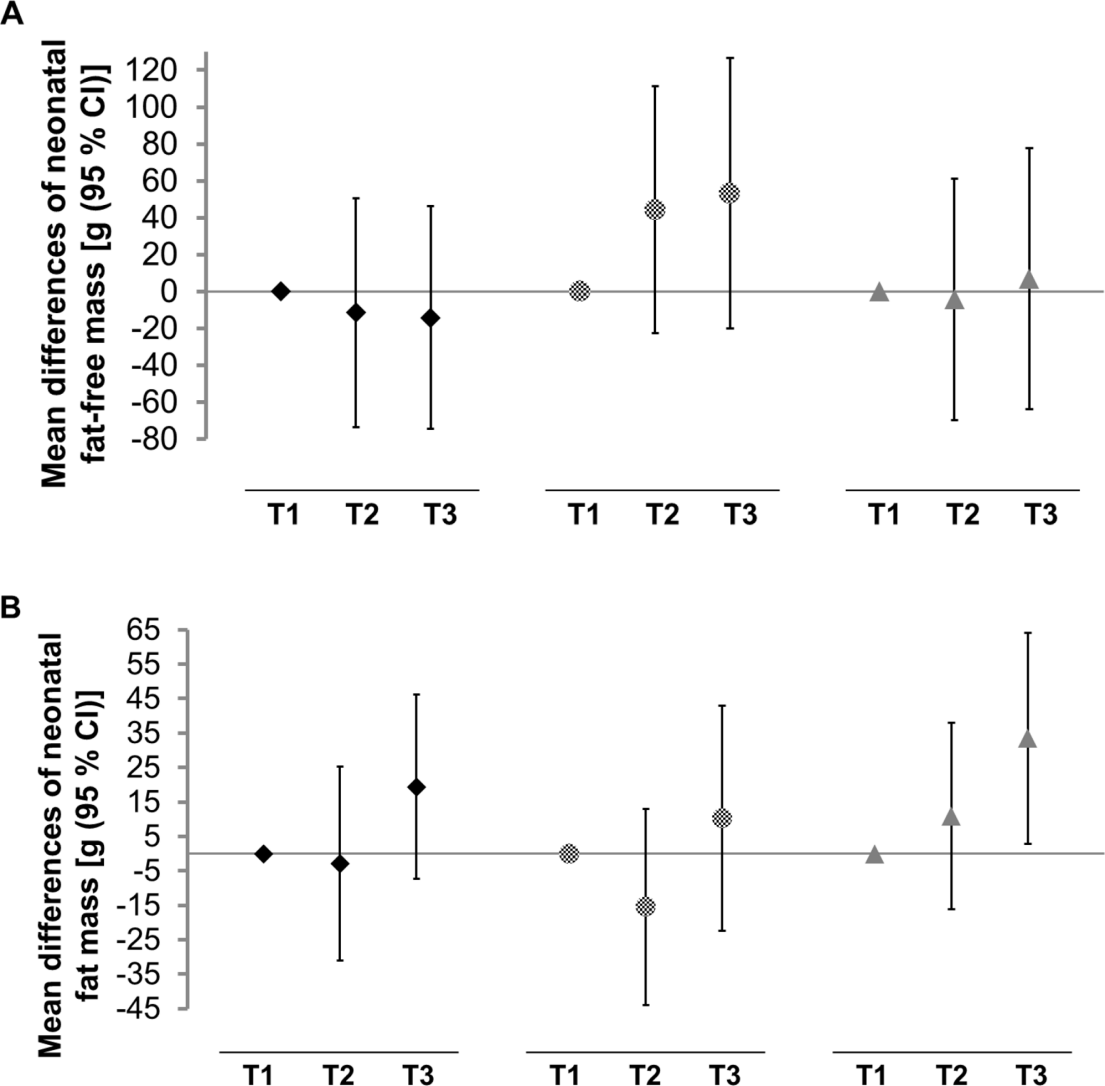
Associations of maternal adherence to the Dietary Diversity Score (♦), Animal-source food pattern () or Vegetarian food pattern (▲) with neonatal absolute (**A**) fat-free mass (g) and (**B**) fat mass (g) among 552 Ethiopian mother-child-pairs. Multiple-adjusted mean differences (β), 95% confidence intervals and *p* values were calculated by linear regression and adjusted for age (mother), sex (child), gestational age, parity, mode of delivery, delivery complications, supplementation and medication during pregnancy, number of antenatal care visits, diseases, Dietary Diversity Score (exploratory dietary patterns only), socioeconomic variables (possession of consumer durables, access to electricity and private piped water, mother’s and father’s occupation and education). Analytical sample (N total = 552) excluded twin births (*n* = 18) and implausible values for Fat-free mass index and Fat mass index (*n* = 74). T = tertile

When applying multiple linear regression, the age- and sex-adjusted birth weight (model 1) was higher among babies born to mothers with the highest DDS tertile compared to those with the lowest DDS tertile (Table 2). However, when adjusting for obstetric history and socioeconomic variables (model 3), the difference was attenuated. No clinically relevant association was found between the mother’s DDS and birth length or gestational age. Similarly, no relevant associations were found between the maternal DDS and neonatal FFM (Table 2; Fig. 2A) and FFMI (Supplementary Fig. 3A, Additional File 1). An increased neonatal FM was seen in the third DDS tertile, when adjusting for maternal age, gestational age, neonatal sex and obstetric history (model 2, Table 2). In model 3, this positive association attenuated but was still discernible [mean difference: 19.4 g (95% CI: -7.4, 46.2)] (Table 2; Fig. 2B). Similarly, babies born to mothers with higher DDS (third tertile) showed an increased FMI compared to babies of mothers with lower DDS (first tertile) (Supplementary Fig. 3B, Additional File 1).

**Table 3:** Associations of exploratory dietary patterns with birth outcomes and body composition among 552 Ethiopian mother-child pairs

	Animal-source food pattern			Vegetarian food pattern		
	Mean difference (β) (95% confidence interval)			Mean difference (β) (95% confidence interval)		
	T1	T2	T3	T1	T2	T3
n	183	185	184	183	185	184
**Gestational age (weeks)**						
Model 1	Ref.	0.03 (-0.22, 0.27)	0.01 (-0.21, 0.23)	Ref.	0.19 (-0.04,0.41)	0.13 (-0.08, 0.35)
Model 2	Ref.	0.02 (-0.22, 0.26)	0.01 (-0.21, 0.23)	Ref.	0.18 (-0.05, 0.41)	0.13 (-0.09, 0.34)
Model 3	Ref.	-0.11 (-0.39, 0.16)	-0.24 (-0.53, 0.05)	Ref.	0.18 (-0.06, 0.42)	0.07 (-0.17, 0.30)
**Birth weight (g)**						
Model 1	Ref.	48.71 (-26.24, 123.67)	90.82 (17.01, 164.63)	Ref.	-5.68 (-87.29, 75.92)	17.43 (-65.22, 100.09)
Model 2	Ref.	39.59 (-35.99, 115.17)	90.75 (16.71, 164.78)	Ref.	-6.43 (-89.07, 76.21)	15.27 (-70.10, 100.64)
Model 3	Ref.	31.34 (-52.47, 115.15)	79.51 (-14.59, 173.61)	Ref.	4.26 (-81.87, 90.40)	42.28 (-52.75, 137.30)
**Length (cm)**						
Model 1	Ref.	0.36 (-0.03, 0.75)	0.42 (0.05, 0.79)	Ref.	-0.13 (-0.53, 0.27)	-0.21 (-0.62, 0.19)
Model 2	Ref.	0.31 (-0.09, 0.71)	0.40 (0.02, 0.77)	Ref.	-0.13 (-0.54, 0.27)	-0.21 (-0.63, 0.21)
Model 3	Ref.	0.26 (-0.19, 0.71)	0.25 (-0.22, 0.73)	Ref.	-0.09 (-0.49, 0.32)	-0.11 (-0.56, 0.33)
**Fat-free mass (g)**						
Model 1	Ref.	53.04 (-4.86, 110.95)	49.75 (-8.19, 107.69)	Ref.	-12.54 (-75.01, 49.92)	-12.95 (-74.89, 48.98)
Model 2	Ref.	44.74 (-14.22, 103.70)	50.11 (-7.20, 107.43)	Ref.	-11.37 (-73.81, 51.07)	-12.77 (-75.96, 50.43)
Model 3	Ref.	44.19 (-22.80, 111.17)	53.14 (-20.26, 126.55)	Ref.	-4.26 (-69.72, 61.20)	6.87 (-63.93, 77.66)
**Fat mass (g)**						
Model 1	Ref.	-5.09 (-29.20, 19.02)	28.44 (4.29, 52.59)	Ref.	11.78 (-13.86, 37.41)	31.53 (3.53, 59.53)
Model 2	Ref.	-5.86 (-30.23, 18.51)	27.29 (3.00, 51.59)	Ref.	9.47 (-16.23, 35.18)	30.04 (1.31, 58.78)
Model 3	Ref.	-15.52 (-43.92, 12.88)	10.32 (-22.34, 42.98)	Ref.	10.82 (-16.23, 37.87)	33.48 (2.82, 64.14)

Multiple-adjusted mean differences (β), 95% confidence intervals and *p* values were calculated by linear regression. Model 1: adjusted for age of the mother, sex of the child and gestational age (for outcome variables other than gestational age); model 2: model 1 + parity, mode of delivery, delivery complications, supplementation and medication during pregnancy, number of antenatal care visits, diseases and Dietary Diversity Score; model 3: model 2 + socioeconomic variables (possession of consumer durables, access to electricity and private piped water, mother’s and father’s occupation and education). Analytical sample (N total = 552) excluded twin births (*n* = 18) and implausible values for Fat-free mass index and Fat mass index (*n* = 74). T = tertile

With regard to the exploratory dietary patterns, compared to those with the lowest AFP adherence (first tertile), mothers with the highest adherence (third tertile) gave birth to babies with higher birth weight (Table 3). In model 3, this difference was only slightly smaller [mean difference: 79.5 (95% CI: -14.6, 173.6)] (Table 3). No relevant associations were found between the mother’s AFP and birth length or gestational age. Regarding absolute FFM, neonates from mothers in the highest tertile of the AFP compared to the lowest tertile showed an increase in absolute FFM across all three adjustment models (Table 3). After adjusting for age of the mother, gestational age, sex of the child, obstetric history, DDS and socioeconomic variables in model 3, the mean difference in neonatal FFM was 53.1 (95% CI: -20.3, 126.6) when comparing mothers with higher adherence to the AFP (third tertile) to those with lower adherence (first tertile) (Table 3; Fig. 2A). Similar positive but weaker tendencies were found between the mother’s adherence to the AFP and neonatal FFMI (Supplementary Fig. 3A, Additional File 1). Concerning absolute FM, a higher maternal adherence to the AFP (third tertile) compared to a lower adherence (first tertile) was related to higher neonatal FM, when adjusting for age of the mother, gestational age and sex of the child (model 1, Table 3). After further adjusting for obstetric history and DDS, this positive association remained (model 2, Table 3) and attenuated after additionally adjusting for socioeconomic variables (model 3, Table 3; Fig. 2B). Very similar results were identified for FMI (Supplementary Fig. 3B, Additional File 1).

With respect to the VFP, compared to those with the lowest adherence (first tertile), mothers with the highest adherence (third tertile) gave birth to slightly heavier babies (model 3, Table 3). Again, there was no relevant association identified between the mother’s VFP and either birth length or gestational age. With regard to body composition, a higher maternal adherence to the VFP (third tertile) compared to a lower adherence (first tertile) was associated with higher neonatal FM, which was identified across all adjustment models (Table 3). In model 3, neonates of mothers with higher adherence to the VFP (third tertile) showed higher absolute FM [mean difference: 33.5 g (95% CI: 2.8, 64.1)] compared to those with lower adherence (first tertile) (Table 3; Fig. 2B). Similarly, a positive association was identified between the VFP and the neonatal FMI (Supplementary Fig. 3B, Additional File 1).

Results of the sensitivity analysis are presented in Supplementary Tables 6 and 7 and described in the Supplementary Statement 3, Additional File 1.

## Discussion

### Summary of main results

The present work investigated associations of maternal dietary exposure in the last week of pregnancy with neonatal body composition in Ethiopian mother-child pairs. Independent of the DDS, maternal adherence to the AFP was associated with higher neonatal FFM and FM. In the adjusted model, both, higher DDS and maternal adherence to the VFP were related to higher neonatal FM but not FFM. While associations of maternal dietary exposure with gestational age and length were of irrelevant magnitude, especially maternal adherence to the AFP was positively associated with birth weight.

### Dietary diversity and neonatal body composition

As previously described, starchy staple foods were most commonly consumed in this Ethiopian population [35–37]. Fish, organ meat and chicken were the least frequently consumed food groups. Although being measured in a comparable way, the median DDS in this study was approximately 2 to 3 score points higher than elsewhere in Ethiopia or other SSA countries, which might be due to the semi-urban and relatively food-secure study area of Jimma Town [36–40].

The relation of maternal diet quality during pregnancy with neonatal body composition has been investigated in high-income countries [33, 41–44], but only one study was conducted in a low-income setting [45]. The most frequently studied pregnancy outcomes in SSA are gestational age, birth weight and length [5–8, 46]. None of these studies calculated the DDS, but rather used the Healthy Eating Index (HEI) [33, 42], which only comprises components reflecting adequacy and moderation.

In ethnically diverse mother-child pairs from Colorado, lower maternal diet quality, as measured by the HEI, was positively associated with infant FM [33]. Similar inverse associations between maternal HEI and neonatal FM were observed in mother-child pairs from Singapore [42]. In contrast, in this study, higher maternal DDS was positively related to neonatal FM. However, existing studies are hardly comparable with the findings of the present work, which is due to methodological disparities. Studies differ in the measurement of exposure e.g. they applied different dietary assessment tools at various time points during pregnancy reflecting different reference periods, and varied in dietary pattern analysis. Moreover, populations of different socio-demographic backgrounds were studied. Different techniques were used to determine neonatal body composition, including anthropometric measures [43, 44], which are not as accurate as ADP to measure body composition [33, 41, 42, 45, 47].

The maternal DDS likely reflects the mother`s nutrient adequacy [21]. The Ethiopian women with a higher DDS consumed red meat, eggs and dairy, dark green leafy vegetables and vitamin A-rich fruits and vegetables more frequently than those with a lower DDS, whose diet was mainly based on starchy foods and legumes. This is in line with previous findings [6, 48]. Animal-source foods, fruits and vegetables represent sources of bioavailable micronutrients, which are essential for the mechanisms of growth or as structural components of body tissue [6, 10, 49]. For example, animal-source foods efficiently supply zinc, which is highly involved in normal growth and development [10, 50, 51]. Green leafy vegetables particularly provide folate [52], which was previously observed to be a potential maternal predictor of greater FM in six-year-old offspring of Indian mother-child pairs [53]. However, the impact of micronutrients on the development and distribution of FFM and FM during the foetal period remains unclear, so that the hypothesised mechanisms are speculative and need further investigation. Moreover, the present study was focused on the overall diet without measurement of single nutrients, which limits any comparison with studies focusing on micronutrients.

### Exploratory dietary patterns and neonatal body composition

With regard to exploratory dietary patterns during pregnancy in relation to neonatal body composition, maternal adherence to a traditional dietary pattern was previously associated with lower neonatal FMI in South-Africans [45]. The traditional dietary pattern was characterised by a high intake of vegetables, beans and legumes, meats and porridge [45]. In a multi-ethnic Asian mother-offspring cohort, adherence to a vegetable, fruit and rice pattern was associated with lower neonatal adiposity [43], while in a second study from the same population, adherence to the vegetable, fruit and rice pattern was associated with higher body fat percentage [44]. In the present study, maternal adherence to the VFP, which was positively correlated with vegetables, fruits, legumes and a roasted grain snack, was related to higher neonatal FM and significantly associated with larger FMI. Again, the results of the studies can hardly be compared, which is due to disparities regarding study population, derived dietary patterns and measurement of neonatal body composition.

Differences in nutrient composition between the exploratory dietary patterns could explain the observed associations. First, the AFP was mainly characterised by high intakes of animal-source foods, which represent a rich source of high-quality proteins (red and organ meat, dairy, eggs and chicken). Adequate protein intake during pregnancy is particularly important for foetal growth and development [54]. Animal-based proteins contain more essential amino acids than plant-based proteins [43, 55], which could possibly drive differences in tissue accretion, such as FFM and FM distribution, during the foetal period [56, 57]. In line with the fact, that animal-source foods have a higher content of bioavailable growth nutrients, the present study identified an increase in FFM among neonates of mothers with higher adherence to the AFP. However, observational studies evaluating the associations between maternal dietary protein and birth outcomes have produced inconsistent results [9, 54].

Second, the frequent intakes of carbohydrate-rich foods in the VFP may contribute to our findings [41]. The quality of maternal carbohydrate intake during pregnancy alters circulating maternal glucose and insulin levels, which influences foetal glucose supply and growth rate [58, 59]. While in Australian mother-infant pairs the quantity of carbohydrate intake during pregnancy was not associated with infants’ body composition [59], the present results imply that the adherence to the VFP promotes foetal fat accretion. However, the described mechanisms are only hypothetical since single nutrients were not measured in this work, and underlying biological mechanisms driving the relationship between maternal dietary nutrients and birth outcomes are not well understood [9, 54].

### Robustness of results

Several sensitivity analyses were conducted to test the robustness of the present findings. Especially excluding mother-child pairs recruited during the lean season seemed to slightly influence the results. However, the changes in effect size were small and precision was low due to the smaller sample size after exclusion. Overall, in this setting, the results seemed to be robust.

### Research prospects and public health implications

This study has important potential implications for future research and public health. It has previously been proposed, that foetal body composition serves as a proxy for the nutritional status of the foetus and represents a major determinant of health status throughout later life [60]. For example, FFM accretion during early infancy has been shown to be positively associated with length at 1 year of age and linear growth from 1 to 5 years of age [61], which potentially drives beneficial short- and long-term consequences like a decreased risk of death throughout infancy and impaired cognitive abilities [1, 32]. On the one hand, neonates with low FM at birth exhibit a higher risk of morbidity in early life and they may also be prone to develop chronic metabolic diseases in later life [15, 62]. On the other hand, an excessive amount of body fat is related to adverse effects on pregnancy outcomes accompanied by an increased susceptibility to later obesity and diseases [63, 64]. However, the present neonates living in a low-income setting were particularly characterised by reduced amounts of FM compared to Western populations [65–67]. Since FM serves as energy storage, its mobilisation is essentially important for survival during times of food insecurity, which is commonly present in low-income countries [15, 68]. Thus, an increased amount of FM of the observed magnitudes may rather be health-beneficial for the neonate. Moreover, the third trimester is the primary period of foetal fat deposition [69], and it is perhaps not surprising that measurements of maternal dietary intake so close to delivery are associated with neonatal adiposity.

### Strengths and limitations

To our knowledge, this is the first study that investigated the effect of maternal dietary diversity and adherence to exploratory dietary patterns in the last week of pregnancy on neonatal body composition in SSA. Still, the results need to be interpreted very cautiously. Due to the secondary objective of the present work, the sample size and power calculation was not based on the objective of this work, which might have driven the low precision of results. The observational nature of the study limits causal inference, because the mother’s diet was retrospectively assessed at the same time of the outcome. The relatively large sample size is outstanding for SSA, but the findings may not be generalisable to the whole SSA population due to the semi-urban context.

Dietary intake was retrospectively obtained from an unvalidated FFQ comprising only 18 food categories, which might have underestimated the true variation in dietary intake [70]. Furthermore, the diet of the last week of pregnancy may not represent the diet throughout the whole pregnancy period, and digestive symptoms at the end of pregnancy as well as cultural aspects influencing e.g. the occurrence of food taboos were not investigated. However, the present work conducted in a semi-urban study area was characterised by a population attending antenatal care visits with relatively high wealth index and dietary diversity. For this reason, it can be speculated, that food taboos only played a minor role in the present work [6, 71]. A main strength of this study is the construction of dietary patterns to consider the complexity of the diet and account for the fact that foods and nutrients are consumed in combination [44, 72]. Not only the hypothesis-driven approach was applied to determine the maternal diversity via calculating the DDS as a valid proxy indicator of nutritional quality [22], but also the data-driven approach was used to exploratory derive maternal dietary patterns by PCA [72].

Another major strength of this study lies in the assessment of neonatal body composition via ADP using the PEA POD^®^, which has previously been validated in infants [18], and comprises several advantages including its non-invasive and fast testing sequence [27]. It has been shown to measure the body composition of infants in a precise, accurate and rapid manner [18].

Instead of excluding participants with incomplete data, the missing variables were filled in using multiple imputation techniques [73]. Several measured covariates and potential confounders were considered when applying regression analysis. For example, the relationship between dietary diversity and birth outcomes seemed to be confounded by socioeconomic conditions. This was further supported by the fact that the socioeconomic status was substantially better in mother-child pairs with higher DDS compared to those with poorer DDS, which is consistent with previous studies [48, 74–76]. Socioeconomic variables were adjusted for in regression analysis. However, residual and unmeasured confounding could not have entirely been excluded. For example, due to non-assessment, pre-gestational or pre-natal anthropometric variables like pre-pregnancy BMI and gestational weight gain could not have been considered. In contrast to residual confounding, overadjustment bias could have attenuated the effect sizes. Overall, the picture was unchanged and results were similar across all adjustment models. Finally, several sensitivity analyses were conducted to test the robustness of the present results.

## Conclusions

In conclusion, the findings of the present work suggest that neonatal body composition is influenced by a diversified diet during pregnancy in this semi-urban Ethiopian study population. While higher maternal intake of animal-source foods was associated with higher neonatal FFM, higher maternal intake of vegetarian foods was associated with higher neonatal FM. Future studies should collect dietary data with more precise instruments and pre-gestational information on maternal anthropometric characteristics. It is currently unknown if the identified alterations in neonatal body composition have positive or negative impacts on long-term body composition and health. Future approaches should further examine postnatal conditions, which potentially outweigh prenatal influences. Therefore, further longitudinal follow-up data on this as well as other cohort studies are necessary to investigate whether neonatal body composition, influenced by maternal nutrition, persists into childhood.

## Electronic supplementary material

Below is the link to the electronic supplementary material.

Supplementary Material 1: Additional File 1 (“Additional_file_1.docx”) includes supplementary material (tables, figures and statements). Within Additional File 1, the supplementary material is ordered in the way it is first mentioned in the article and referenced explicitly by file name within the body of the article.

## Data Availability

The iABC Study is part of the Jimma University Clinical and Nutrition (JUCAN) Research Partnership, Jimma, Ethiopia (https://www.ju.edu.et/jucan/). The iABC study data can be made available upon request directed to the JUCAN Steering Committee currently headed by Dr. Melkamu Berhane (melkamuberhane@yahoo.com). Data cannot be made available in a public repository due to legal and ethical restraints. The informed consent for the iABC study was collected in 2008–2011 and public sharing of any individual level data was not part of the consent at that time.

## Abbreviations

ADP: Air-displacement plethysmography
AFP: Animal-source food pattern
BMI: Body mass index
CI: Confidence interval
DDS: Dietary Diversity Score
FAO: Food and Agricultural Organisation of the United Nations
FCS: Fully conditional specification
FFM: Fat-free mass
FFMI: Fat-free mass index
FFQ: Food frequency questionnaire
FM: Fat mass
FMI: Fat mass index
iABC: Infant anthropometry and body composition study
ID: Identification number
IQR: Interquartile range
IWI: International Wealth Index by Smits and Steendijk [77]
SD: Standard deviation
VFP: Vegetarian food pattern

## References

[1] Black RE, Victora CG, Walker SP, Bhutta ZA, Christian P, de Onis M, et al. Maternal and child undernutrition and overweight in low-income and middle-income countries. Lancet. 2013;382(9890):427–51. 23746772 10.1016/S0140-6736(13)60937-X

[2] Wells JC, Sawaya AL, Wibaek R, Mwangome M, Poullas MS, Yajnik CS, et al. The double burden of malnutrition: aetiological pathways and consequences for health. Lancet. 2020;395(10217):75–88. 31852605 10.1016/S0140-6736(19)32472-9 PMC7613491

[3] Cetin I, Cardellicchio M. Physiology of Pregnancy: Interaction between Mother and Child. Annales Nestlé (English ed). 2010;68(1):7–15.

[4] Lartey A. Maternal and child nutrition in Sub-saharan Africa: challenges and interventions. Proc Nutr Soc. 2008;67(1):105–8. 18234138 10.1017/S0029665108006083

[5] Chia AR, Chen LW, Lai JS, Wong CH, Neelakantan N, van Dam RM, et al. Maternal dietary patterns and birth outcomes: a systematic review and Meta-analysis. Adv Nutr. 2019;10(4):685–95. 31041446 10.1093/advances/nmy123 PMC6628847

[6] Zerfu TA, Umeta M, Baye K. Dietary diversity during pregnancy is associated with reduced risk of maternal anemia, preterm delivery, and low birth weight in a prospective cohort study in rural Ethiopia. Am J Clin Nutr. 2016;103(6):1482–8. 27169832 10.3945/ajcn.115.116798

[7] Saaka M. Maternal dietary diversity and infant outcome of pregnant women in Northern Ghana. Int J Child Health Nutr. 2012;1:148–56.

[8] Abubakari A, Jahn A. Maternal dietary patterns and practices and Birth Weight in Northern Ghana. PLoS ONE. 2016;11(9):e0162285. 27611597 10.1371/journal.pone.0162285 PMC5017622

[9] Abu-Saad K, Fraser D. Maternal nutrition and birth outcomes. Epidemiol Rev. 2010;32:5–25. 20237078 10.1093/epirev/mxq001

[10] Fall CH, Yajnik CS, Rao S, Davies AA, Brown N, Farrant HJ. Micronutrients and fetal growth. J Nutr. 2003;133(5 Suppl 2):S1747–56. 10.1093/jn/133.5.1747S 12730494

[11] Sánchez-Villegas A, Brito N, Doreste-Alonso J, Nissensohn M, Henriquez P, Hermoso M, et al. Methodological aspects of the study of dietary patterns during pregnancy and maternal and infant health outcomes. A systematic review. Matern Child Nutr. 2010;6(Suppl 2):100–11. 22296253 10.1111/j.1740-8709.2010.00263.x PMC6860860

[12] Chen X, Zhao D, Mao X, Xia Y, Baker PN, Zhang H. Maternal dietary patterns and pregnancy outcome. Nutrients. 2016;8(6). 10.3390/nu8060351 PMC4924192 27338455

[13] Hu FB. Dietary pattern analysis: a new direction in nutritional epidemiology. Curr Opin Lipidol. 2002;13(1):3–9. 11790957 10.1097/00041433-200202000-00002

[14] Cespedes EM, Hu FB. Dietary patterns: from nutritional epidemiologic analysis to national guidelines. Am J Clin Nutr. 2015;101(5):899–900. 25832336 10.3945/ajcn.115.110213 PMC4409695

[15] Carberry AE, Raynes-Greenow CH, Turner RM, Askie LM, Jeffery HE. Is body fat percentage a better measure of undernutrition in newborns than birth weight percentiles? Pediatr Res. 2013;74(6):730–6. 24002331 10.1038/pr.2013.156

[16] Wells JC. A critique of the expression of paediatric body composition data. Arch Dis Child. 2001;85(1):67–72. 11420208 10.1136/adc.85.1.67 PMC1718830

[17] Wells JC. Toward body composition reference data for infants, children, and adolescents. Adv Nutr. 2014;5(3):S320–9. 10.3945/an.113.005371 PMC4013189 24829484

[18] Andersen GS, Girma T, Wells JC, Kaestel P, Leventi M, Hother AL, et al. Body composition from birth to 6 mo of age in Ethiopian infants: reference data obtained by air-displacement plethysmography. Am J Clin Nutr. 2013;98(4):885–94. 23985805 10.3945/ajcn.113.063032

[19] Andersen GS, Girma T, Wells JC, Kaestel P, Michaelsen KF, Friis H. Fat and fat-free mass at birth: air displacement plethysmography measurements on 350 Ethiopian newborns. Pediatr Res. 2011;70(5):501–6. 21772228 10.1203/PDR.0b013e31822d7470

[20] Wibaek R, Vistisen D, Girma T, Admassu B, Abera M, Abdissa A, et al. Associations of fat mass and fat-free mass accretion in infancy with body composition and cardiometabolic risk markers at 5 years: the Ethiopian iABC birth cohort study. PLoS Med. 2019;16(8):e1002888. 31430287 10.1371/journal.pmed.1002888 PMC6701744

[21] Kennedy G, Ballard T, Dop M. Guidelines for Measuring Household and Individual Dietary Diversity. Rome, Italy: Food and Agriculture Organization of the United Nations (FAO); 2011.

[22] Arimond M, Wiesmann D, Becquey E, Carriquiry A, Daniels MC, Deitchler M, et al. Simple food group diversity indicators predict micronutrient adequacy of women’s diets in 5 diverse, resource-poor settings. J Nutr. 2010;140(11):S2059–69. 10.3945/jn.110.123414 PMC2955880 20881077

[23] Zhao J, Li Z, Gao Q, Zhao H, Chen S, Huang L, et al. A review of statistical methods for dietary pattern analysis. Nutr J. 2021;20(1):37. 33874970 10.1186/s12937-021-00692-7 PMC8056502

[24] Ballard JL, Novak KK, Driver M. A simplified score for assessment of fetal maturation of newly born infants. J Pediatr. 1979;95(5 Pt 1):769–74. 490248 10.1016/s0022-3476(79)80734-9

[25] Ballard JL, Khoury JC, Wedig K, Wang L, Eilers-Walsman BL, Lipp R. New Ballard Score, expanded to include extremely premature infants. J Pediatr. 1991;119(3):417–23. 1880657 10.1016/s0022-3476(05)82056-6

[26] Andersen GS. Body composition in Ethiopian infants: air-displacement plethysmography from birth to six months of age [Ph.D]. Denmark: University of Copenhagen; 2011.

[27] Urlando A, Dempster P, Aitkens S. A new air displacement plethysmograph for the measurement of body composition in infants. Pediatr Res. 2003;53(3):486–92. 12595599 10.1203/01.PDR.0000049669.74793.E3

[28] Dempster P, Aitkens S. A new air displacement method for the determination of human body composition. Med Sci Sports Exerc. 1995;27(12):1692–7. 8614327

[29] Ethiopian Public Health Institute. Ethiopia National Food Consumption Survey. Ethiopia; 2013.

[30] O’Rourke N, Hatcher L. A Step-by-Step Approach to Using SAS^®^ for Factor Analysis and Structural Equation Modeling. Cary, NC: SAS Institute Inc.; 2013.

[31] Frank LK, Kroger J, Schulze MB, Bedu-Addo G, Mockenhaupt FP, Danquah I. Dietary patterns in urban Ghana and risk of type 2 diabetes. Br J Nutr. 2014;112(1):89–98. 24708913 10.1017/S000711451400052X

[32] United Nations Children’s Fund (UNICEF). UNICEF’s approach to scaling up nutrition for mothers and their children. Discussion paper. Programme Division, UNICEF, New York 2015.

[33] Shapiro AL, Kaar JL, Crume TL, Starling AP, Siega-Riz AM, Ringham BM, et al. Maternal diet quality in pregnancy and neonatal adiposity: the healthy start study. Int J Obes (Lond). 2016;40(7):1056–62. 27133623 10.1038/ijo.2016.79 PMC5356926

[34] Danquah I, Galbete C, Meeks K, Nicolaou M, Klipstein-Grobusch K, Addo J, et al. Food variety, dietary diversity, and type 2 diabetes in a multi-center cross-sectional study among Ghanaian migrants in Europe and their compatriots in Ghana: the RODAM study. Eur J Nutr. 2018;57(8):2723–33. 28948398 10.1007/s00394-017-1538-4 PMC6267387

[35] Zerfu TA, Baye K, Faber M. Dietary diversity cutoff values predicting anemia varied between mid and term of pregnancy: a prospective cohort study. J Health Popul Nutr. 2019;38(1):44. 31836026 10.1186/s41043-019-0196-y PMC6911293

[36] Aliwo S, Fentie M, Awoke T, Gizaw Z. Dietary diversity practice and associated factors among pregnant women in North East Ethiopia. BMC Res Notes. 2019;12(1):123. 30845950 10.1186/s13104-019-4159-6 PMC6407270

[37] Weldehaweria NB, Misgina KH, Weldu MG, Gebregiorgis YS, Gebrezgi BH, Zewdie SW, et al. Dietary diversity and related factors among lactating women visiting public health facilities in Aksum town, Tigray, Northern Ethiopia. BMC Nutr. 2016;2(1):38.

[38] Leroy JL, Bliznashka DKO, Ruel L. Tubaramure, a Food-Assisted Maternal and Child Health and Nutrition Program in Burundi, Increased Household Food Security and Energy and Micronutrient Consumption, and maternal and child dietary diversity: a cluster-randomized controlled trial. J Nutr. 2020;150(4):945–57. 31858128 10.1093/jn/nxz295 PMC7138675

[39] Saaka M, Oladele J, Larbi A, Hoeschle-Zeledon I. Dietary diversity is not Associated with Haematological Status of pregnant women Resident in Rural areas of Northern Ghana. J Nutr Metab. 2017;2017:8497892. 28168052 10.1155/2017/8497892 PMC5267082

[40] Yeneabat T, Adugna H, Asmamaw T, Wubetu M, Admas M, Hailu G, et al. Maternal dietary diversity and micronutrient adequacy during pregnancy and related factors in East Gojjam Zone, Northwest Ethiopia, 2016. BMC Pregnancy Childbirth. 2019;19(1):173. 31092223 10.1186/s12884-019-2299-2 PMC6521398

[41] Starling AP, Sauder KA, Kaar JL, Shapiro AL, Siega-Riz AM, Dabelea D. Maternal dietary patterns during pregnancy are Associated with Newborn Body Composition. J Nutr. 2017;147(7):1334–9. 28539412 10.3945/jn.117.248948 PMC5483965

[42] Chia AR, Tint MT, Han CY, Chen LW, Colega M, Aris IM, et al. Adherence to a healthy eating index for pregnant women is associated with lower neonatal adiposity in a multiethnic Asian cohort: the growing up in Singapore towards healthy outcomes (GUSTO) study. Am J Clin Nutr. 2018;107(1):71–9. 29381790 10.1093/ajcn/nqx003 PMC5972656

[43] Chen LW, Aris IM, Bernard JY, Tint MT, Chia A, Colega M et al. Associations of maternal dietary patterns during pregnancy with offspring adiposity from Birth until 54 months of age. Nutrients. 2016;9(1). 10.3390/nu9010002 PMC5295046 28025503

[44] Chia AR, de Seymour JV, Colega M, Chen LW, Chan YH, Aris IM, et al. A vegetable, fruit, and white rice dietary pattern during pregnancy is associated with a lower risk of preterm birth and larger birth size in a multiethnic Asian cohort: the growing up in Singapore towards healthy outcomes (GUSTO) cohort study. Am J Clin Nutr. 2016;104(5):1416–23. 27733407 10.3945/ajcn.116.133892

[45] Wrottesley SV, Ong KK, Pisa PT, Norris SA. Maternal traditional dietary pattern and antiretroviral treatment exposure are associated with neonatal size and adiposity in urban, black South africans. Br J Nutr. 2018;120(5):557–66. 30058507 10.1017/S0007114518001708 PMC6773599

[46] Madzorera I, Isanaka S, Wang M, Msamanga GI, Urassa W, Hertzmark E, et al. Maternal dietary diversity and dietary quality scores in relation to adverse birth outcomes in Tanzanian women. Am J Clin Nutr. 2020;112(3):695–706. 32651998 10.1093/ajcn/nqaa172 PMC7458779

[47] Wiechers C, Kirchhof S, Maas C, Poets CF, Franz AR. Neonatal body composition by air displacement plethysmography in healthy term singletons: a systematic review. BMC Pediatr. 2019;19(1):489. 31830946 10.1186/s12887-019-1867-y PMC6907141

[48] Workicho A, Belachew T, Feyissa GT, Wondafrash B, Lachat C, Verstraeten R, et al. Household dietary diversity and animal source food consumption in Ethiopia: evidence from the 2011 Welfare Monitoring Survey. BMC Public Health. 2016;16(1):1192. 27884138 10.1186/s12889-016-3861-8 PMC5123272

[49] Horan MK, McGowan CA, Gibney ER, Donnelly JM, McAuliffe FM. The association between maternal dietary micronutrient intake and neonatal anthropometry - secondary analysis from the ROLO study. Nutr J. 2015;14:105. 26445882 10.1186/s12937-015-0095-z PMC4597429

[50] Zhang Z, Goldsmith PD, Winter-Nelson A. The Importance of Animal Source Foods for Nutrient Sufficiency in the developing world: the Zambia scenario. Food Nutr Bull. 2016;37(3):303–16. 10.1177/0379572116647823 27150300

[51] Shah D, Sachdev HP. Effect of gestational zinc deficiency on pregnancy outcomes: summary of observation studies and zinc supplementation trials. Br J Nutr. 2001;85(Suppl 2):S101–8. 11509097 10.1079/bjn2000301

[52] Rao S, Yajnik CS, Kanade A, Fall CH, Margetts BM, Jackson AA, et al. Intake of micronutrient-rich foods in rural Indian mothers is associated with the size of their babies at birth: Pune maternal Nutrition Study. J Nutr. 2001;131(4):1217–24. 11285330 10.1093/jn/131.4.1217

[53] Yajnik CS, Deshpande SS, Jackson AA, Refsum H, Rao S, Fisher DJ, et al. Vitamin B12 and folate concentrations during pregnancy and insulin resistance in the offspring: the Pune maternal Nutrition Study. Diabetologia. 2008;51(1):29–38. 17851649 10.1007/s00125-007-0793-y PMC2100429

[54] Chong MF, Chia AR, Colega M, Tint MT, Aris IM, Chong YS, et al. Maternal protein intake during pregnancy is not Associated with offspring birth weight in a multiethnic Asian Population. J Nutr. 2015;145(6):1303–10. 25948786 10.3945/jn.114.205948

[55] van Vliet S, Burd NA, van Loon LJ. The Skeletal Muscle Anabolic Response to plant- versus animal-based protein consumption. J Nutr. 2015;145(9):1981–91. 26224750 10.3945/jn.114.204305

[56] Heiman ML, Greenway FL. A healthy gastrointestinal microbiome is dependent on dietary diversity. Mol Metab. 2016;5(5):317–20. 27110483 10.1016/j.molmet.2016.02.005 PMC4837298

[57] García-Mantrana I, Bertua B, Martínez-Costa C, Collado MC. Perinatal nutrition: how to take care of the gut microbiota? Clin Nutr Exp. 2016;6:3–16.

[58] Clapp JF 3. Maternal carbohydrate intake and pregnancy outcome. Proc Nutr Soc. 2002;61(1):45–50. 10.1079/pns2001129 12008645

[59] McKenzie KM, Dissanayake HU, McMullan R, Caterson ID, Celermajer DS, Gordon A et al. Quantity and quality of carbohydrate intake during pregnancy, newborn body fatness and Cardiac Autonomic Control. Conferred Cardiovasc Risk? Nutrients. 2017;9(12). 10.3390/nu9121375 PMC5748825 29257088

[60] Toro-Ramos T, Paley C, Pi-Sunyer FX, Gallagher D. Body composition during fetal development and infancy through the age of 5 years. Eur J Clin Nutr. 2015;69(12):1279–89. 26242725 10.1038/ejcn.2015.117 PMC4680980

[61] Admassu B, Ritz C, Wells JCK, Girma T, Andersen GS, Belachew T, et al. Accretion of Fat-Free Mass Rather Than Fat Mass in Infancy is positively Associated with Linear Growth in Childhood. J Nutr. 2018;148(4):607–15. 29659955 10.1093/jn/nxy003

[62] Barker DJ, Eriksson JG, Forsén T, Osmond C. Fetal origins of adult disease: strength of effects and biological basis. Int J Epidemiol. 2002;31(6):1235–9. 12540728 10.1093/ije/31.6.1235

[63] Kizirian NV, Markovic TP, Muirhead R, Brodie S, Garnett SP, Louie JC et al. Macronutrient Balance and Dietary Glycemic Index in pregnancy predict neonatal body composition. Nutrients. 2016;8(5). 10.3390/nu8050270 PMC4882683 27164136

[64] Yu ZB, Han SP, Zhu GZ, Zhu C, Wang XJ, Cao XG, et al. Birth weight and subsequent risk of obesity: a systematic review and meta-analysis. Obes Rev. 2011;12(7):525–42. 21438992 10.1111/j.1467-789X.2011.00867.x

[65] Carberry AE, Colditz PB, Lingwood BE. Body composition from birth to 4.5 months in infants born to non-obese women. Pediatr Res. 2010;68(1):84–8. 20351656 10.1203/PDR.0b013e3181df5421

[66] Crume TL, Shapiro AL, Brinton JT, Glueck DH, Martinez M, Kohn M, et al. Maternal fuels and metabolic measures during pregnancy and neonatal body composition: the healthy start study. J Clin Endocrinol Metab. 2015;100(4):1672–80. 25574704 10.1210/jc.2014-2949 PMC4399301

[67] Crume TL, Brinton JT, Shapiro A, Kaar J, Glueck DH, Siega-Riz AM, et al. Maternal dietary intake during pregnancy and offspring body composition: the healthy start study. Am J Obstet Gynecol. 2016;215(5):609. e1- e8. 10.1016/j.ajog.2016.06.035 PMC5571832 27371352

[68] Grijalva-Eternod CS, Wells JC, Girma T, Kaestel P, Admassu B, Friis H, et al. Midupper arm circumference and weight-for-length z scores have different associations with body composition: evidence from a cohort of Ethiopian infants. Am J Clin Nutr. 2015;102(3):593–9. 26224296 10.3945/ajcn.114.106419

[69] Ziegler EE, O’Donnell AM, Nelson SE, Fomon SJ. Body composition of the reference fetus. Growth. 1976;40(4):329–41. 1010389

[70] Naska A, Lagiou A, Lagiou P. Dietary assessment methods in epidemiological research: current state of the art and future prospects. F1000Res. 2017;6:926. 28690835 10.12688/f1000research.10703.1 PMC5482335

[71] Daba G, Beyene F, Fekadu H, Garoma W. Assessment of knowledge of pregnant mothers on Maternal Nutrition and Associated Factors in Guto Gida Woreda, East Wollega Zone, Ethiopia. J Nutr Food Sci. 2013;3(6):235.

[72] Smith AD, Emmett PM, Newby PK, Northstone K. Dietary patterns obtained through principal components analysis: the effect of input variable quantification. Br J Nutr. 2013;109(10):1881–91. 22950853 10.1017/S0007114512003868

[73] He Y. Missing data analysis using multiple imputation: getting to the heart of the matter. Circ Cardiovasc Qual Outcomes. 2010;3(1):98–105. 20123676 10.1161/CIRCOUTCOMES.109.875658 PMC2818781

[74] Morseth MS, Grewal NK, Kaasa IS, Hatloy A, Barikmo I, Henjum S. Dietary diversity is related to socioeconomic status among adult saharawi refugees living in Algeria. BMC Public Health. 2017;17(1):621. 28673263 10.1186/s12889-017-4527-x PMC5496305

[75] Girma W, Genebo T. Determinants of nutritional status of women and children in Ethiopia. Maryland, USA: Ethiopia Health and Nutrition Research Institute; 2002.

[76] Savy M, Martin-Prevel Y, Traissac P, Delpeuch F. Measuring dietary diversity in rural Burkina Faso: comparison of a 1-day and a 3-day dietary recall. Public Health Nutr. 2007;10(1):71–8. 17212836 10.1017/S1368980007219627

[77] Smits J, Steendijk R. The International Wealth Index (IWI). Soc Indic Res. 2015;122(1):65–85.

