# Supplementary material for: Maternal dietary patterns as predictors of neonatal body composition in Ethiopia: the IABC birth cohort study
Source: BMC Pregnancy Childbirth. 2025 Apr 2;25:386. doi: 10.1186/s12884-025-07256-1 (PMC11967154; doi:10.1186/s12884-025-07256-1)
Supplement: Supplementary file 1 — Supplementary Material 1: Additional File 1 (“Additional_file_1.docx”) includes supplementary material (tables, figures and statements). Within Additional File 1, the supplementary material is ordered in the way it is first mentioned in the article and referenced explicitly by file name within the body of the article. [file 12884_2025_7256_MOESM1_ESM.docx]

Table of contents

[Supplementary Figure 1 2](#_Toc127351348)

[Supplementary Table 1 3](#_Toc127351349)

[Supplementary Table 2 4](#_Toc127351350)

[Supplementary Table 3 5](#_Toc127351351)

[Supplementary Statement 1 6](#_Toc127351352)

[Supplementary Statement 2 7](#_Toc127351353)

[Supplementary Figure 2 8](#_Toc127351354)

[Supplementary Table 4 9](#_Toc127351355)

[Supplementary Table 5 10](#_Toc127351356)

[Supplementary Figure 3 11](#_Toc127351357)

[Supplementary Table 6 12](#_Toc127351358)

[Supplementary Table 7 13](#_Toc127351359)

[Supplementary Statement 3 14](#_Toc127351360)

[References 14](#_Toc127351361)

**
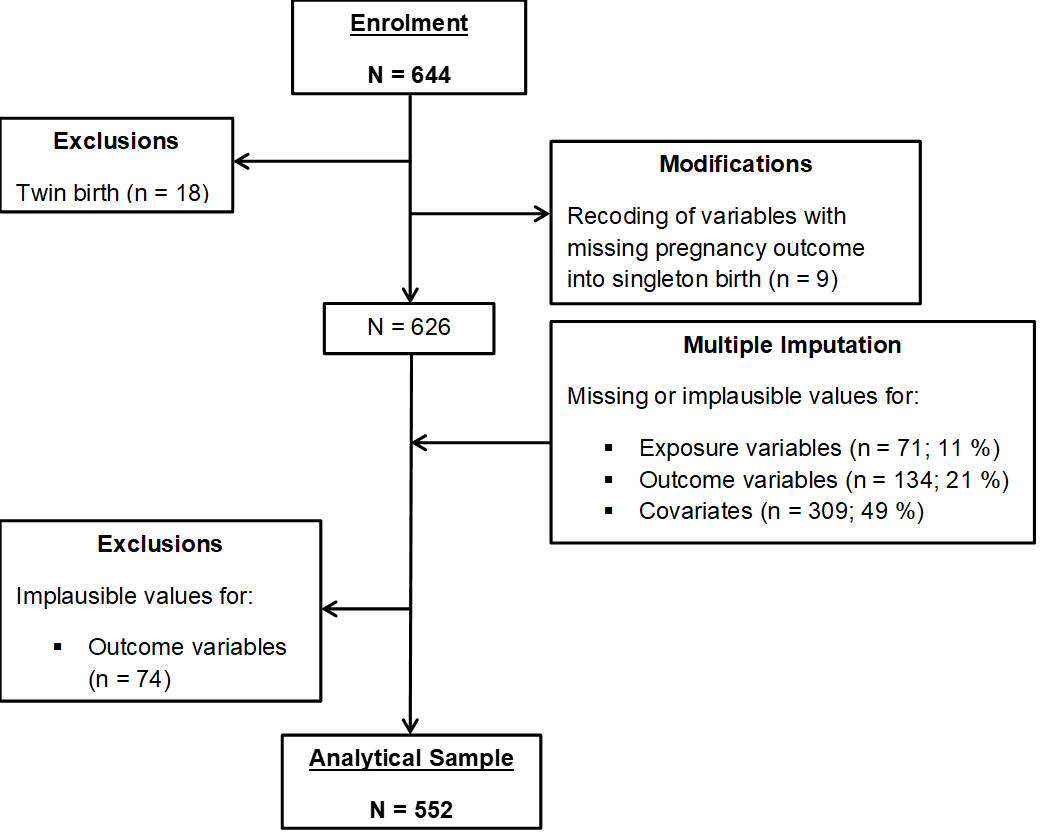
**

Supplementary Figure 1 Flow diagram of excluded participants and analytical study population in the iABC-1 study.

Supplementary Table 1 Aggregation of food groups based on the food frequency questionnaire (FFQ) in the iABC-1 study.

| **No.** | **FFQ Food Items** | **No.** | **DDS Categories** | **No.** | **Food Groups for PCA** |
| --- | --- | --- | --- | --- | --- |
| 1 | *Injera* (teff) | 1 | Starchy staples  (Cereals, roots and tubers) | 1 | *Injera* (flat, thin bread) |
| 2 | *Injera* (maize) |  |  |  |  |
| 3 | *Injera* (mixed) |  |  |  |  |
| 4 | Bread, rice, spaghetti |  |  | 2 | Bread, rice, spaghetti |
| 5 | *Kolo* |  |  | 3 | *Kolo* (grain snack) |
| 6 | Lentils, beans, chickpeas, peas (including as part of *wot*) | 2 | Legumes, nuts and seeds | 4 | Lentils, beans, chickpeas, peas |
| 7 | Chicken (including *doro* *wot)* | 3 | Meat, poultry and fish | 5 | Chicken |
| 8 | Red meat, e.g. beef, goat, sheep (including as part of *wot)* |  |  | 6 | Red meat, e.g. beef, goat, sheep |
| 9 | Fish (including as part of *wot)* |  |  | 7 | Fish |
| 10 | Organ meats (liver, heart, kidney) (including as part of *wot)* | 4 | Organ meat | 8 | Organ meats (liver, heart, kidney) |
| 11 | Dairy products, e.g. milk, yoghurt, cheese | 5 | Milk and milk products | 9 | Dairy products, e.g. milk, yoghurt, cheese |
| 12 | Eggs (including as part of *wot)* | 6 | Eggs | 10 | Eggs |
| 13 | Green leafy vegetables, e.g. kale (including as part of *wot)* | 7 | Green leafy vegetables | 11 | Green leafy vegetables, e.g. kale |
| 14 | Carrots (including as part of *wot)* | 8 | Other vitamin A fruits and vegetables | 12 | Carrots |
| 15 | Mango, papaya (include juice) |  |  | 13 | Fruits |
| 16 | Avocado (including juice) | 9 | Other Fruits |  |  |
| 17 | Orange, lemon |  |  |  |  |
| 18 | Banana, pineapple |  |  |  |  |

Aggregation of food groups to be included in the calculation of the Dietary Diversity Score (DDS) and the principal component analysis (PCA) based on the FFQ in the iABC-1 cohort study. No. = number.

Supplementary Table 2 Socioeconomic characteristics collected in the iABC-1 cohort study and used in this work.

|  | **Categories** |
| --- | --- |
| Mother’s and spouse’s occupation | - Farmer - Public employee - Private employee - Merchant - Unemployed - Day worker - Student - Only mother: housewife; small scale traders - Only spouse: retired - Other |
| Mother’s and spouse’s education | - No school - Some primary school - Completed primary school - Secondary school - Higher education |
| Type of house | - Tukul - Mud house - Brick/block house - Mud and brick house |
| Type of toilet facility | - Open field - Shared pit latrine - Private pit latrine - WC - Other |
| Source of drinking water | - Private pipe water - Public pipe water - Protected spring - Unprotected spring - River - Well - Other |
| Access to electricity | - Yes / No |
| Possession of household items (yes/no) | - Radio - Television - Telephone - Mobile phone - Refrigerator - Electric stove - Electric mitad - Bicycle - Motor cycle - Car |

Supplementary Table 3 Proportion of missingness in the iABC-1 cohort study (N = 626).

| **Variable** | **%** | **Variable** | **%** |
| --- | --- | --- | --- |
| **ID** | **0.00** | Parity | 2.40 |
| **Date of interview** | **0.00** | Neonatal weight-for-length z-score | 2.40 |
| **Date of birth** | **0.00** | Supplementation | 3.19 |
| Gestational age | 0.00 | Medication | 3.19 |
| Sex of the child | 0.00 | Fish intake | 3.35 |
| Neonatal body mass index *z*-score | 0.32 | Banana, pineapple intake | 3.35 |
| Neonatal length | 0.32 | Red meats, e.g. beef, goat sheep intake | 3.42 |
| Neonatal birth weight | 0.32 | Paternal occupation | 3.51 |
| Neonatal length-for-age z-score | 0.32 | Injera (Mixed) intake | 3.51 |
| Neonatal weight-for-age z-score | 0.32 | Lentils, beans, chick peas, peas intake | 3.51 |
| Neonatal ponderal index | 0.32 | Dairy products, e.g. milk, yoghurt, cheese intake | 3.51 |
| Neonatal body mass index | 0.32 | Chicken intake | 3.67 |
| Maternal occupation | 1.12 | Organ meats intake | 3.67 |
| Religion | 1.28 | Green leafy vegetables, e.g. kale intake | 3.67 |
| Ethnicity | 1.28 | Meals | 3.83 |
| Maternal education | 1.28 | Avocado intake | 3.83 |
| Marital Status | 1.28 | Orange, lemon intake | 3.83 |
| Living status | 1.44 | Paternal education | 3.99 |
| Number of rooms in the house | 1.44 | Mango, papaya intake | 3.99 |
| Mode of delivery | 1.44 | Injera (Teff) intake | 4.15 |
| Neonatal triceps skinfold | 1.44 | Injera (Maize) intake | 4.15 |
| Neonatal head circumference | 1.44 | Bread, Rice, Spaghetti intake | 4.15 |
| Neonatal abdominal circumference | 1.44 | Kolo intake | 4.15 |
| Neonatal arm circumference | 1.44 | Delivery complications | 4.15 |
| Type of house | 1.60 | Carrots intake | 4.47 |
| Possession of domestic animals | 1.60 | Eggs intake | 4.79 |
| Neonatal subscapular skinfold | 1.60 | Antenatal care (yes/no) | 5.59 |
| Source of drinking water | 1.76 | Fat-free mass (kg)* | 7.19 |
| Access to electricity | 1.76 | Antenatal care (place of registration) | 12.30 |
| Possession of radio | 1.76 | Diseases | 12.62 |
| Source of water (not for drinking) | 1.92 | Antenatal care (amount of visits) | 12.62 |
| Possession of cooking facilities | 2.08 | Fat mass (kg)* | 14.70 |
| Possession of mobile phone | 2.08 | Maternal triceps skinfold | 18.37 |
| Possession of refrigerator | 2.08 | Maternal mid-upper arm circumference | 18.53 |
| Possession of electric stove | 2.08 | Maternal subscapular skinfold | 19.97 |
| Possession of electric mitad | 2.08 | Maternal weight | 26.84 |
| Possession of bicycle | 2.08 | Maternal body mass index | 27.48 |
| Maternal age | 2.24 | Maternal height | 27.80 |
| Type of toilet facility | 2.24 |  |  |
| Possession of TV | 2.24 |  |  |
| Possession of motor cycle (moto) | 2.24 |  |  |
| Possession of car | 2.24 |  |  |
| Possession of telephone | 2.40 |  |  |

*****Absolute FM and FFM were out of the reference range in 92 (15 %) and 45 (7 %) neonates in the 626 mother-child pairs (1), and assumed to be implausible being thus recoded into missing values before presenting the proportion of missingness. Variables which were not included in the imputation model are highlighted in bold.

Supplementary Statement 1 Multiple imputation used to handle missing data.

The proportion of missing information highly varied across the variables (0.0 % to 27.8 %). It was assumed, that missing data depended on information, which was already observed, and was thus missing at random. Although this assumption cannot be proven, it is more likely to be plausible that data are missing at random when more variables are included in the multiple imputation model (2). All variables used in the subsequent analyses and possible auxiliary variables were included in the imputation process. Categorical variables were imputed using the discriminant fully conditional specification method, which is also known as multiple imputation by chained equations. This method uses separate conditional univariate imputation models specified for each incomplete variable, with other variables as predictors. The imputed values of one variable are further used to impute the next variable, thereafter repeating this process until convergence is reached (3, 4). Continuous variables were imputed using the predictive mean matching method, which means that missing data are imputed using the observed values with the closest predictive mean from a linear regression model (3). The Statistical Analysis System (SAS) procedure PROC MI was used to perform the multiple imputation.

Since the relative efficiency of the imputation was high (> 98 %) and the fraction missing information was low (< 8 %), the imputed dataset was based on five imputations. According to Schafer, there seems to be little or no practical benefit of using more than five to ten imputations (5). As with ten imputations the relative efficiency did not dramatically change and the fraction of missingness even increased, five imputations were applied to generate the imputed dataset for subsequent analyses. Moreover, fat-mass index and fat-free mass index were excluded from the imputation, since their inclusion produced lower relative efficiency and a higher proportion of fraction missing information.

Following the multiple imputation, the distributions of the imputed variables were compared to the distribution of the variables prior to imputation. Since the distributions and descriptive statistics were very similar between the raw and imputed variables, the generated imputed dataset with five imputations was used for further statistical analyses. In SAS the PROC MIANALYZE procedure was used to produce pooled estimates.

Supplementary Statement 2 Sensitivity analysis methods.

Several sensitivity analyses were conducted to consider the effects of the mother’s energy intake using maternal BMI as a proxy, the international wealth index (IWI) and seasonality.

According to Smits and Steendijk, the IWI was calculated from data on seven consumer durables (possession of a TV, refrigerator, phone, bicycle, car, cheap and expensive utensil), access to two public services (clean water, electricity) and one housing characteristic (toilet facility) (6). The variables cheap utensils and expensive utensils are constructed variables. A cheap utensil was defined as having a price below 50 USD, which, in this analysis, was only assumed to be true for the radio. Instead, an expensive utensil was defined to cost roughly over 300 USD, including the motorcycle, car, TV, refrigerator, telephone, mobile phone, bicycle, electric stove and electric mitad (6). A mother-child pair who owned a radio, received the value ‘1’ for the variable ‘cheap utensils’. Similarly, the mother-child pair was coded as ‘1’ for ‘expensive utensils’, if it possessed at least one expensive item. Since there were no elements of medium toilet facility quality collected via the questionnaire, the asset ‘quality of toilet facility’ only comprised two categories. The original IWI further included two more housing characteristics (number of sleeping rooms and quality of floor material), which were not measured in this study. Nevertheless, it was still possible to compute the IWI by using an adapted formula supplied by the authors (7). The IWI scale ranges from 0 to 100, with a higher value indicating a better household’s economic situation (6).

Different seasons in Jimma, southwest Ethiopia, were determined and distinguished between the harvest or food-sufficient season from October to December and the pre-harvest or lean season from June to August (8). Multiple linear regression analysis was performed with a sample excluding participants recruited in the pre-harvest or lean season from June to August (n = 417) (8). Overall, the results where compared to model 3 of the main association results.


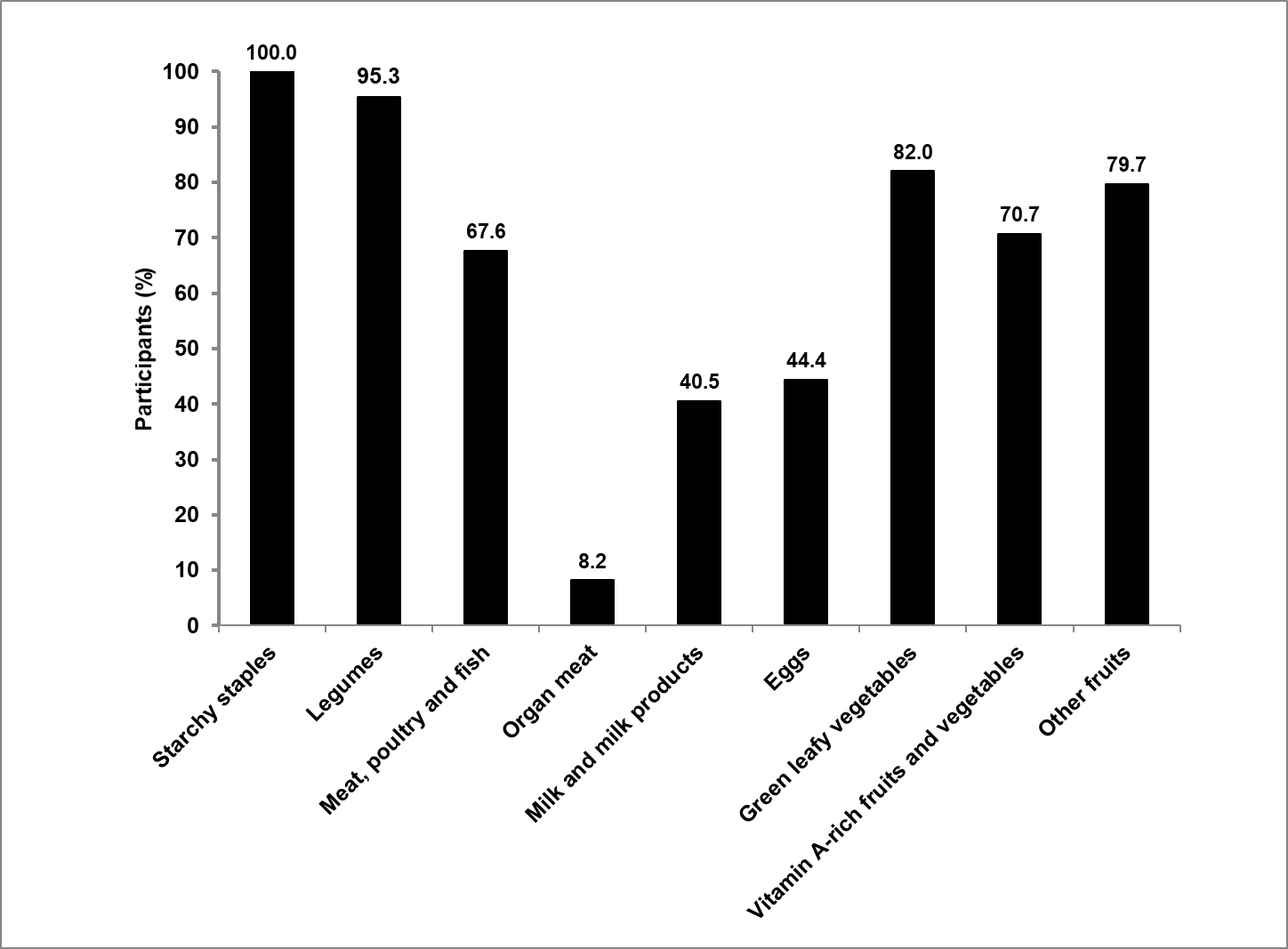


Supplementary Figure 2 Percentage of pregnant Ethiopian women meeting the scoring criteria of the Dietary Diversity Score food groups.

Supplementary Table 4 General parental characteristics across tertiles (T) of exploratory dietary patterns among 552 Ethiopian pregnant women.

|  | **Animal-source food pattern** | | | **Vegetarian food pattern** | | |
| --- | --- | --- | --- | --- | --- | --- |
|  | **T1** | **T2** | **T3** | **T1** | **T2** | **T3** |
| n | 183 | 185 | 184 | 183 | 185 | 184 |
| Pattern Score (mean ± SD) | -0.89 ± 0.23 | -0.21 ± 0.22 | 1.10 ± 0.92 | -1.02 ± 0.33 | -0.10 ± 0.24 | 1.12 ± 0.72 |
| DDS [median (interquartile range)] | 5 (4 - 6) | 6 (5 - 7) | 7 (6 - 8) | 6 (4 - 6) | 6 (5 - 7) | 6 (5 - 7) |
| Mother’s age (years; mean ± SD) | 24.1 ± 5.0 | 23.8 ± 4.3 | 24.5 ± 4.6 | 23.7 ± 4.7 | 24.4 ± 4.7 | 24.3 ± 4.4 |
| Mother’s BMI (kg/m^2^; mean ± SD) | 22.0 ± 2.6 | 22.7 ± 2.9 | 23.0 ± 3.3 | 22.8 ± 3.1 | 22.5 ± 2.9 | 22.4 ± 3.0 |
| **Obstetric history** |  |  |  |  |  |  |
| Antenatal care (yes) | 90.8 | 94.6 | 94.7 | 91.4 | 94.3 | 94.5 |
| Parity (≥ 3) | 23.1 | 21.8 | 20.9 | 19.4 | 22.6 | 23.8 |
| Spontaneous vertex delivery (yes) | 92.9 | 93.5 | 89.8 | 91.2 | 91.1 | 93.9 |
| Delivery complications (no) | 95.7 | 94.5 | 92.0 | 94.1 | 94.7 | 93.4 |
| Diseases (yes) | 4.4 | 5.0 | 3.3 | 6.6 | 1.5 | 4.6 |
| Supplementation (yes) | 10.0 | 16.4 | 15.3 | 15.7 | 16.7 | 9.3 |
| Medication (yes) | 11.7 | 16.0 | 18.7 | 20.5 | 12.2 | 13.8 |
| **Religion** |  |  |  |  |  |  |
| Muslim | 42.0 | 42.8 | 48.1 | 52.7 | 37.9 | 42.3 |
| Orthodox Christianity | 39.8 | 41.4 | 32.2 | 33.0 | 43.1 | 37.3 |
| **Mother’s education** |  |  |  |  |  |  |
| Higher education | 7.7 | 13.3 | 20.8 | 13.9 | 11.9 | 16.1 |
| **Father’s education** |  |  |  |  |  |  |
| Higher education | 9.2 | 24.2 | 28.4 | 17.6 | 21.3 | 23.0 |
| **Mother’s occupation** |  |  |  |  |  |  |
| Employee (private and public) | 12.0 | 21.6 | 27.2 | 19.8 | 17.3 | 23.9 |
| **Father’s occupation** |  |  |  |  |  |  |
| Employee (private and public) | 53.4 | 63.2 | 61.9 | 59.0 | 58.4 | 61.3 |
| **Consumer durables** |  |  |  |  |  |  |
| Access to electricity (yes) | 92.8 | 95.6 | 97.0 | 95.0 | 96.4 | 93.9 |
| Access to private piped water (yes) | 48.0 | 60.2 | 74.4 | 63.3 | 55.5 | 63.9 |
| Possession of household items (> 5) | 6.5 | 11.1 | 23.5 | 13.5 | 13.9 | 13.7 |
| International wealth index (mean ± SD) | 44.3 ± 17.3 | 51.9 ± 17.6 | 58.6 ± 17.0 | 53.3 ± 18.3 | 50.1 ± 18.7 | 51.5 ± 17.6 |

Data were shown as relative frequencies (%), unless otherwise stated. Analytical sample (N total = 552) excluded twin births (n = 18) and implausible values for Fat-free mass index and Fat mass index (n = 74). SD = standard deviation, DDS = Dietary Diversity Score, BMI = Body Mass Index.

Supplementary Table 5 Characteristics and body composition of 552 Ethiopian newborns across tertiles (T) of exploratory dietary patterns.

|  | **Animal-source food pattern** | | | **Vegetarian food pattern** | | |
| --- | --- | --- | --- | --- | --- | --- |
|  | **T1** | **T2** | **T3** | **T1** | **T2** | **T3** |
| n | 183 | 185 | 184 | 183 | 185 | 184 |
| Gestational age (weeks) | 39.0 ± 1.1 | 39.0 ± 1.0 | 39.0 ± 1.0 | 38.9 ± 1.1 | 39.1 ± 1.0 | 39.1 ± 1.0 |
| Sex of the child (male; %) | 51.4 | 50.9 | 43.5 | 45.2 | 50.7 | 49.9 |
| **Anthropometric characteristics** |  |  |  |  |  |  |
| Birth weight (g) | 3050.6 ± 354.6 | 3099.4 ± 344.5 | 3136.6 ± 382.9 | 3079.2 ± 379.3 | 3094.7 ± 362.2 | 3112.9 ± 345.2 |
| Length (cm) | 49.1 ± 1.9 | 49.5 ± 1.7 | 49.5 ± 1.8 | 49.4 ± 1.8 | 49.4 ± 1.9 | 49.3 ± 1.8 |
| **Body composition** |  |  |  |  |  |  |
| Fat-free mass (g) | 2813.8 ± 276.4 | 2866.5 ± 279.5 | 2856.8 ± 285.2 | 2844.2 ± 291.1 | 2848.6 ± 279.9 | 2844.5 ± 272.8 |
| Fat-free mass index (kg/m^2^) | 11.6 ± 0.7 | 11.7 ± 0.7 | 11.6 ± 0.8 | 11.6 ± 0.8 | 11.7 ± 0.7 | 11.7 ± 0.7 |
| Fat mass (g) | 228.1 ± 121.6 | 223.3 ± 112.7 | 258.7 ± 120.3 | 220.7 ± 114.1 | 235.3 ± 120.7 | 254.1 ± 120.6 |
| Fat mass index (kg/m^2^) | 0.9 ± 0.5 | 0.9 ± 0.4 | 1.1 ± 0.5 | 0.9 ± 0.4 | 1.0 ± 0.5 | 1.0 ± 0.5 |

Data were shown as mean ± standard deviation, unless otherwise stated. Analytical sample (N total = 552) excluded twin births (n = 18) and implausible values for Fat-free mass index and Fat mass index (n = 74).


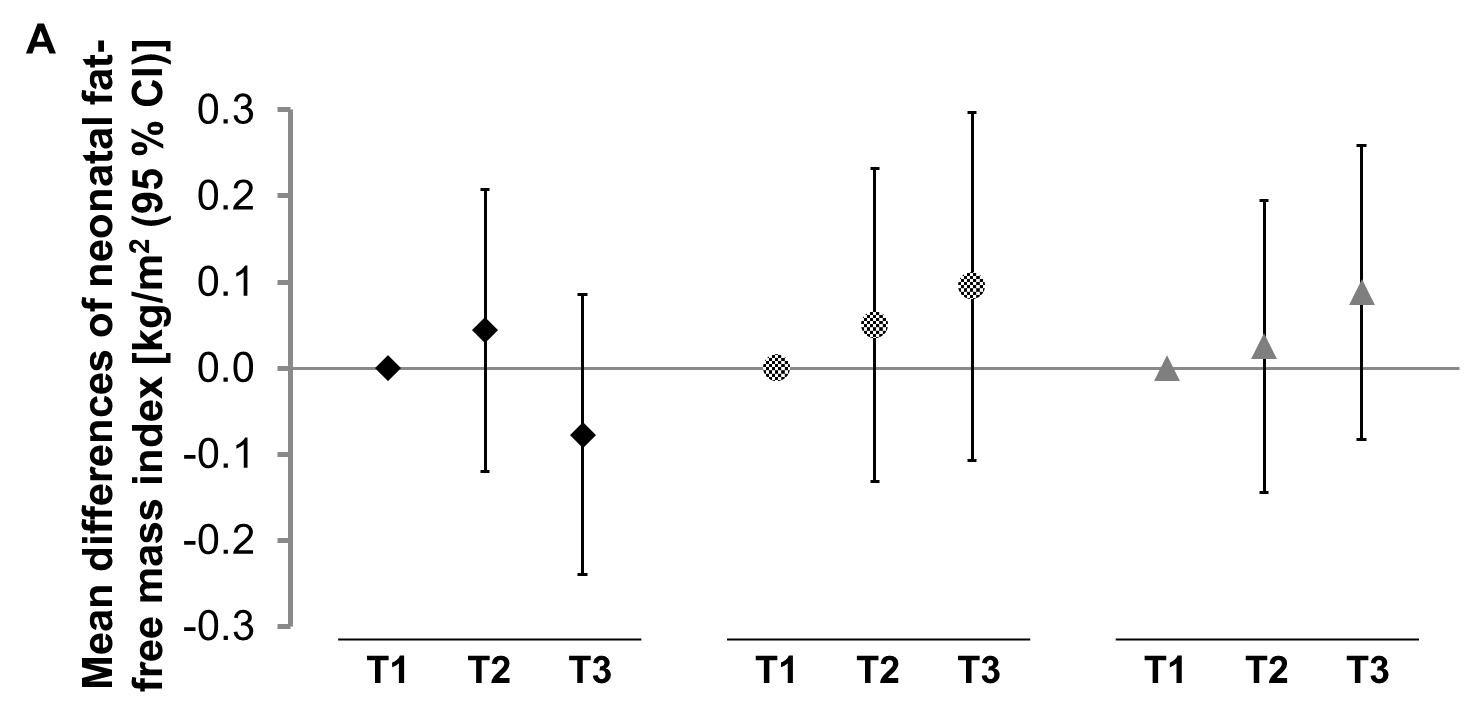


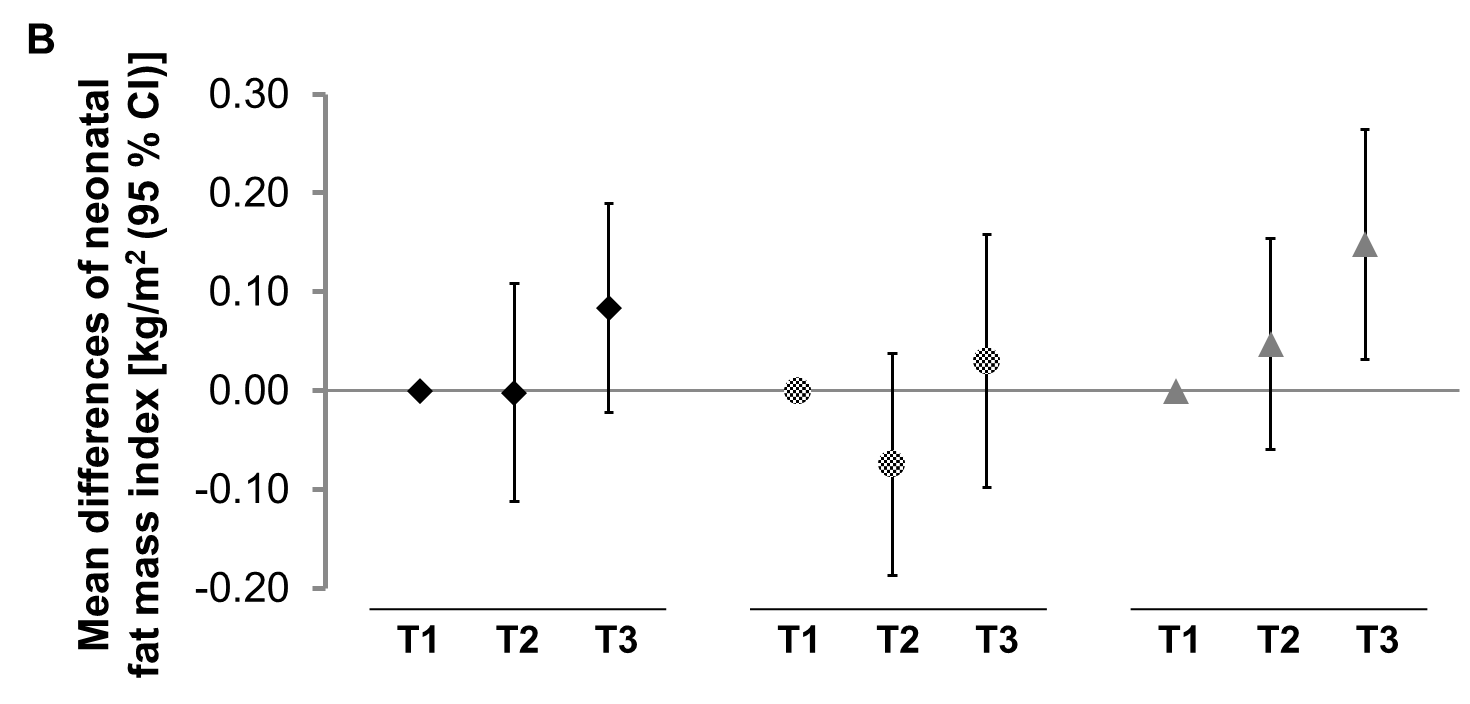


Supplementary Figure 3 Associations of maternal adherence to the Dietary Diversity Score (♦), Animal-source food pattern () or Vegetarian food pattern (▲) with neonatal (A) fat-free mass index (kg/m²) and (B) fat mass index (kg/m²) among 552 Ethiopian mother-child-pairs. Multiple-adjusted mean differences (β), 95 % confidence intervals and *p* values were calculated by linear regression and adjusted for age (mother), sex (child), gestational age, parity, mode of delivery, delivery complications, supplementation and medication during pregnancy, number of antenatal care visits, diseases, Dietary Diversity Score (exploratory dietary patterns only), socioeconomic variables (possession of consumer durables, access to electricity and private piped water, mother’s and father’s occupation and education). Analytical sample (N total = 552) excluded twin births (n = 18) and implausible values for Fat-free mass index and Fat mass index (n = 74). T = tertile.

Supplementary Table 6 Multiple-adjusted associations of maternal Dietary Diversity Score with neonatal body composition among 552 Ethiopian mother-child-pairs.

|  | **Dietary Diversity Score** | | |
| --- | --- | --- | --- |
|  | **Mean difference (β) (95% confidence interval)** | | |
|  | **T1** | **T2** | **T3** |
| n | 210 | 152 | 190 |
| **Fat-free mass (g)** |  |  |  |
| Multiple-adjusted model | Ref. | -11.53 (-73.71, 50.65) | -14.13 (-74.69, 46.43) |
| + BMI | Ref. | -11.64 (-74.03, 50.74) | -15.70 (-76.51, 45.11) |
| + IWI | Ref. | -6.89 (-67.55, 53.78) | -11.78 (-71.11, 47.55) |
| Lean season excluded | Ref. | 15.83 (-55.75, 87.41) | -29.11 (-97.01, 38.79) |
| **Fat mass (g)** |  |  |  |
| Multiple-adjusted model | Ref. | -2.90 (-31.11, 25.31) | 19.40 (-7.38, 46.18) |
| + BMI | Ref. | -2.97 (-31.25, 25.31) | 19.14 (-7.61, 45.89) |
| + IWI | Ref. | -3.25 (-31.19, 24.69) | 21.36 (-4.36, 47.08) |
| Lean season excluded | Ref. | 18.26 (-13.47, 49.98) | 23.88 (-6.68, 54.44) |

Multiple-adjusted mean differences (β), 95 % confidence intervals and *p* values were calculated by linear regression and adjusted for maternal age, gestational age, sex of the child, parity, mode of delivery, delivery complications, supplementation and medication during pregnancy, number of antenatal care visits, diseases, socioeconomic variables (possession of consumer durables, access to electricity and private piped water, mother’s and father’s occupation and education); + BMI: additionally adjusted for body mass index (BMI) of the mother. + IWI: adjusted for the International Wealth Index (IWI) instead of single socioeconomic variables. Analytical sample (N total = 552) excluded twin births (n = 18) and implausible values for Fat-free mass index and Fat mass index (n = 74). Moreover, participants recruited in the lean season (n=135) were excluded. T = tertile.

Supplementary Table 7 Associations of exploratory dietary patterns with birth outcomes and body composition among 552 Ethiopian mother-child pairs.

|  | **Animal-source food pattern** | | | **Vegetarian food pattern** | | |
| --- | --- | --- | --- | --- | --- | --- |
|  | **Mean difference (β) (95% confidence interval)** | | | **Mean difference (β) (95% confidence interval)** | | |
|  | **T1** | **T2** | **T3** | **T1** | **T2** | **T3** |
| n | 183 | 185 | 184 | 183 | 185 | 184 |
| **Fat-free mass (g)** |  |  |  |  |  |  |
| Multiple-adjusted model | Ref. | 44.19 (-22.80, 111.17) | 53.14 (-20.26, 126.55) | Ref. | -4.26 (-69.72, 61.20) | 6.87 (-63.93, 77.66) |
| + BMI | Ref. | 41.87 (-25.97, 109.71) | 51.73 (-22.41, 125.88) | Ref. | -3.26 (-67.81, 61.29) | 8.99 (-61.13, 79.11) |
| + IWI | Ref. | 47.69 (-18.54, 113.92) | 55.18 (-16.31, 126.67) | Ref. | -4.52 (-69.19, 60.15) | -5.74 (-73.30, 61.82) |
| Lean season excluded | Ref. | 35.79 (-50.61, 122.20) | 45.07 (-45.00, 135.14) | Ref. | -5.69 (-84.10, 72.72) | 2.94 (-72.15, 78.03) |
| **Fat mass (g)** |  |  |  |  |  |  |
| Multiple-adjusted model | Ref. | -15.52 (-43.92, 12.88) | 10.32 (-22.34, 42.98) | Ref. | 10.82 (-16.23, 37.87) | 33.48 (2.82, 64.14) |
| + BMI | Ref. | -15.89 (-44.31, 12.53) | 10.08 (-22.47, 42.64) | Ref. | 11.09 (-15.75, 37.92) | 34.03 (4.13, 63.93) |
| + IWI | Ref. | -17.13 (-44.10, 9.85) | 6.54 (-24.93, 38.00) | Ref. | 9.73 (-16.33, 35.78) | 29.54 (-0.02, 59.10) |
| Lean season excluded | Ref. | -19.49 (-51.96, 12.98) | -0.24 (-37.64, 37.16) | Ref. | 14.69 (-16.24, 45.61) | 38.94 (3.79, 74.08) |

Multiple-adjusted mean differences (β), 95 % confidence intervals and *p* values were calculated by linear regression and adjusted for maternal age, gestational age, sex of the child, parity, mode of delivery, delivery complications, supplementation and medication during pregnancy, number of antenatal care visits, diseases, dietary diversity index, socioeconomic variables (possession of consumer durables, access to electricity and private piped water, mother’s and father’s occupation and education); + BMI: additionally adjusted for body mass index (BMI) of the mother. + IWI: adjusted for the International Wealth Index (IWI) instead of single socioeconomic variables. Analytical sample (N total = 552) excluded twin births (n = 18) and implausible values for Fat-free mass index and Fat mass index (n = 74). Moreover, participants recruited in the lean season (n=135) were excluded. T = tertile.

Supplementary Statement 3 Results of sensitivity analysis.

Supplementary Table 4 and 5 illustrate the results of the sensitivity analysis. The association between maternal DDS and neonatal FFM or FM hardly changed when the mother’s BMI or the IWI were considered (Supplementary Table 6). However, considering seasonality slightly increased the association between maternal DDS and neonatal body composition, especially regarding FFM (Supplementary Table 6). With respect to the maternal adherence to either AFP or VFP, excluding mother-child pairs recruited during the lean season resulted in slightly changed effect estimates. For example, the association between maternal adherence to the AFP and neonatal body composition was attenuated, while the association between maternal adherence to the VFP and FM was slightly stronger (Supplementary Table 7).

# References

1. Andersen GS, Girma T, Wells JC, Kaestel P, Leventi M, Hother AL, et al. Body composition from birth to 6 mo of age in Ethiopian infants: reference data obtained by air-displacement plethysmography. Am J Clin Nutr. 2013;98(4):885-94.

2. Pedersen AB, Mikkelsen EM, Cronin-Fenton D, Kristensen NR, Pham TM, Pedersen L, et al. Missing data and multiple imputation in clinical epidemiological research. Clin Epidemiol. 2017;9:157-66.

3. Hayati Rezvan P, Lee KJ, Simpson JA. The rise of multiple imputation: a review of the reporting and implementation of the method in medical research. BMC Med Res Methodol. 2015;15:30.

4. He Y. Missing data analysis using multiple imputation: getting to the heart of the matter. Circ Cardiovasc Qual Outcomes. 2010;3(1):98-105.

5. Schafer JL. Multiple imputation: a primer. Stat Methods Med Res. 1999;8(1):3-15.

6. Smits J, Steendijk R. The International Wealth Index (IWI). Social Indicators Research. 2015;122(1):65-85.

7. Smits J, Steendijk R. International Wealth Index (IWI) The Netherlands: Global Data Lab; [Available from: <https://globaldatalab.org/iwi/downloads/>.]

8. Wondafrash M, Huybregts L, Lachat C, Bouckaert KP, Kolsteren P. Dietary diversity predicts dietary quality regardless of season in 6-12-month-old infants in south-west Ethiopia. Public Health Nutr. 2016;19(14):2485-94.
